# The Role of Spirituality and Religiosity in the Maintenance and Recovery of Psychosis: A Systematic Review

**DOI:** 10.1111/eip.70061

**Published:** 2025-07-03

**Authors:** Megan Westhead, Anna Georgiades

**Affiliations:** ^1^ Department of Psychosis Studies Institute of Psychiatry, Psychology, and Neuroscience (IoPPN), King's College London London UK; ^2^ Brent Early Intervention Service, CNWL, NHS Foundation Trust London UK

**Keywords:** cognitive model, maintenance, psychosis, recovery, religiosity/spirituality

## Abstract

**Objective:**

Many individuals with psychosis consider themselves religious or spiritual and report using religion as a means of coping with their illness. However, research exploring the impact of religiosity and spirituality on the experience of psychosis is sparse, with most studies focusing on delusions or hallucinations with religious content.

**Methods:**

A systematic review examined the evidence regarding the role of religiosity/spirituality in the maintenance and recovery of psychosis.

**Results:**

A total of 35 studies were eligible for inclusion. In terms of maintenance, religiosity and spirituality were positively correlated with positive symptoms of psychosis. Individualised religious practice was associated with more severe delusions, while high intrinsic religiosity was associated with an increased severity of auditory and visual hallucinations. In terms of recovery, Positive Religious Coping (PRC) was found to improve wellbeing, quality of life, treatment expectancy, and medication adherence, while Negative Religious Coping (NRC) increased suicidality, positive symptom severity, and illness duration, and reduced social functioning. Holding religious/spiritual explanatory models was correlated with increased psychosis symptom severity and delayed recovery, while holding a bio‐psychosocial explanatory model assisted with recovery.

**Conclusions:**

Religiosity/spirituality appears to play a significant role in the maintenance and recovery of positive symptoms of psychosis. CBTp could be enhanced by integrating religiosity and spirituality into assessment, formulation, and the development of targeted interventions. This approach would promote more culturally adapted CBTp and improved engagement with clients from diverse cultural backgrounds in Early Intervention Services.

## 1 Introduction

Religiosity and spirituality are complex, multidimensional concepts encompassing religious affiliation and community, religious/spiritual practices and beliefs, as well as religious and spiritual coping (Charzyńska 2015). Specifically, religion embodies a system of beliefs and rituals, which encourages closeness to the transcendent and reflects the extent to which a person engages in these practices (Cohen et al. 2010; Koenig 2009; Koenig 2012; Mohr et al. 2011; Robles‐García et al. 2014; Röhricht et al. 2009). Spirituality pertains to existential questions regarding one's meaning and life purpose, often viewing oneself as part of a wider spiritual or cosmic force (Charzyńska 2015; Cohen et al. 2010; Mohr et al. 2011; Piedmont 1999; Robles‐García et al. 2014; Tanyi 2002). Despite religion holding primarily social characteristics and spirituality more individualised characteristics (Koenig 2009), these terms are often used interchangeably and are deemed markers of an individual's belief system, functioning, and self‐conceptualisation (Röhricht et al. 2009). For many people, religion and spirituality are fundamental aspects of life, which inevitably shape one's experience of stress and mental health problems. Many individuals with psychosis consider themselves religious and turn to their faith as a way to cope with the distress caused by their symptoms (Kroll and Sheehan 1989; Neeleman and Lewis 1994; Tepper et al. 2001). However, research about the impact of religiosity/spirituality on the experience of psychosis is limited, with most studies focusing on delusions or hallucinations with religious content (Cook 2015). Indeed, spirituality/religiosity have been associated with poorer outcomes, including increased risk of experiencing religious delusions (Anderson‐Schmidt et al. 2019; Getz et al. 2001; Huang et al. 2011; Siddle et al. 2002) and a longer illness duration (Huang et al. 2011). However, other studies have found that spirituality/religiosity in psychosis can be beneficial in regard to improving quality of life (QoL) (Caqueo‐Urizar et al. 2016; Cohen et al. 2010; Huguelet et al. 2016; Roystonn et al. 2021; Serfaty et al. 2020; Shah et al. 2011; Triveni et al. 2017), reducing positive symptom severity (Esan and Lawal 2021; Triveni et al. 2017), reducing suicide risk (Huguelet et al. 2007), and increasing medication adherence (Borras et al. 2007), thereby emphasising their clinical significance. Thus, a systematic review exploring the role of spirituality and religiosity in the maintenance and recovery of psychosis would aid in the development of a novel *Cognitive Model of Spirituality and Religiosity in Psychosis*, which would enhance culturally adapted Cognitive Behavioural Therapy for Psychosis (CBTp) practices. An improved understanding of the role of religiosity/spirituality in psychosis would enhance engagement with services and would foster the development of more personalised and symptom‐specific formulations, hopefully leading to improved outcomes in psychosis.

### 1.1 Aims of the Study

This systematic review aimed to synthesise the available literature regarding the role of religiosity and spirituality in the maintenance and recovery of psychosis, with the view to proposing cognitive models of spirituality/religiosity in psychosis and the development of socratic questions to facilitate CBTp assessment in this domain.

## 2 Method

The PRISMA guidelines were adhered to (Page et al. 2021). The inclusion criteria and methods were specified in advance of the search being conducted and were registered with PROSPERO (registration number: CRD42024549926).

### 2.1 Search Strategy

The databases searched for the present systematic review included: APA PsycINFO, EMBASE, Global Health, and MEDLINE (including PubMed) from database onset to 30th June 2024. The following search strings were used: Psychosis OR Psychotic OR Schizophreni* AND Spiritual* OR Religio* OR Religious coping OR Spiritual coping AND Delusion* OR Hallucination* OR Positive symptom* OR Negative symptom* OR Religious delusion* OR Psychotic experience* OR Psychotic symptom* AND Onset OR Recovery OR Relaps* OR Hospitali* OR Rehospitali* OR Adherence OR Quality of life OR Treatment response OR Treatment seeking OR Wellbeing OR Suicide.

### 2.2 Study Selection

**FIGURE 1:**
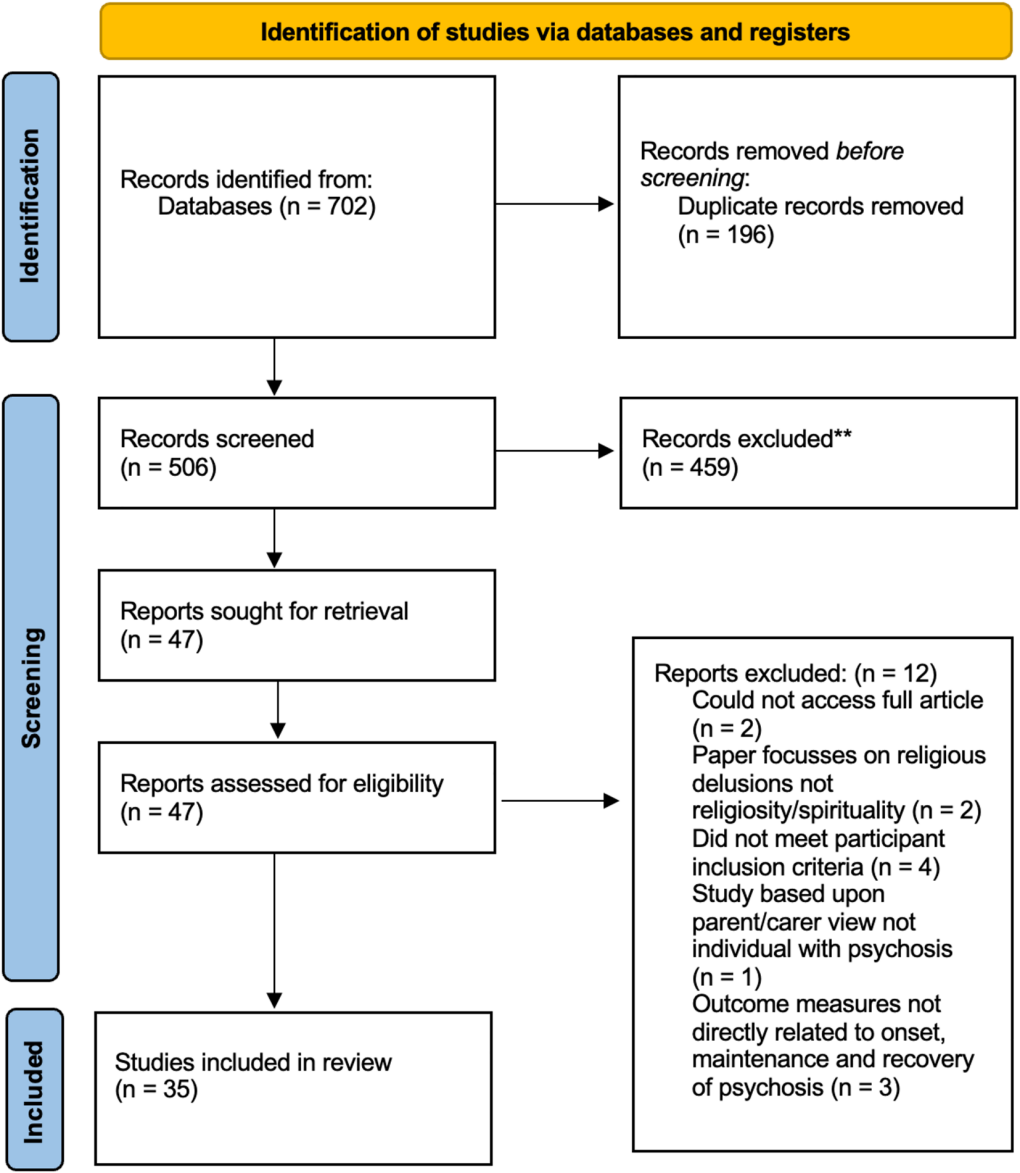
PRISMA 2020 flow diagram.

All types of studies were considered for inclusion. Eligible studies needed to (a) explore the role of religiosity and/or spirituality in the maintenance and/or recovery of psychosis, and/or (b) explore the role of religiosity/spirituality on mood and/or functioning in psychosis. Studies were eligible for inclusion if they were written in English, published in peer‐reviewed journals, and included participants with a psychosis disorder as measured using a reliable psychometric tool (e.g., International Classification of Diseases, eleventh edition, or ICD‐11, World Health Organization 2019; Diagnostic and Statistical Manual of Mental Disorders, fifth edition, or DSM‐5, American Psychiatric Association 2013). The PRISMA flow diagram details the study selection process (see Figure 1).

### 2.3 Exclusion Criteria

Case studies, reviews, meta‐analyses, unpublished/grey literature, and those written in languages other than English were excluded from this review. Studies investigating participants with organic psychosis or drug‐induced psychosis were also excluded. Moreover, studies were excluded if they did not investigate an association between religiosity/spirituality and psychosis.

### 2.4 Data Extraction Process

The following characteristics of all included studies were detailed within an excel spreadsheet following de‐duplication: author and year, country, sample size, mean age, diagnostic tools/questionnaires, results and clinical findings. The screening process was conducted by two reviewers (M.W and A.G).

### 2.5 Quality Appraisal

Quality ratings were determined using the following tools: EPHPP tool (Thomas 2003) for quantitative studies (see Table 1) and the JBI Critical Appraisal Checklist for Qualitative Research (Joanna Briggs Institute; Lockwood et al. 2015) (see Table 2).

**TABLE 1:** Quality assessment for quantitative studies‐EPHPP tool.

Author (Year)	Selection bias	Study design	Confounders	Data collection methods	Withdrawals and dropouts	Analyses (appropriateness)	Global rating
AbdelGawad et al. (2017)	Moderate	Moderate	Strong	Strong	Moderate	Yes	Strong
Amadi et al. (2021)	Moderate	Moderate	Moderate	Strong	Strong	Yes	Strong
Anderson‐Schmidt et al. (2019)	Strong	Moderate	Strong	Moderate	Strong	Yes	Strong
Borras et al. (2007)	Moderate	Moderate	Strong	Strong	Strong	Yes	Strong
Brito et al. (2021)	Strong	Moderate	Strong	Moderate	Moderate	Yes	Strong
Caqueo‐Urízar et al. (2015)	Moderate	Moderate	Strong	Strong	Moderate	Yes	Strong
Caqueo‐Urizar et al. (2016)	Strong	Moderate	Strong	Strong	Moderate	Yes	Strong
Cohen et al. (2010)	Moderate	Moderate	Strong	Strong	Moderate	Yes	Strong
Compton and Furman (2005)	Weak	Moderate	Weak	Moderate	Moderate	Yes	Weak
Esan and Lawal (2021)	Moderate	Moderate	Strong	Strong	Moderate	Yes	Strong
Fares et al. (2023)	Moderate	Moderate	Strong	Moderate	Moderate	Yes	Strong
Getz et al. (2001)	Moderate	Moderate	Strong	Strong	Moderate	Yes	Strong
Huang et al. (2011)	Moderate	Moderate	Strong	Moderate	Moderate	Yes	Strong
Huguelet et al. (1997)	Moderate	Moderate	Strong	Weak	Weak	Yes	Weak
Huguelet et al. (2007)	Moderate	Moderate	Moderate	Moderate	Strong	Yes	Strong
Huguelet et al. (2016)	Moderate	Moderate	Strong	Strong	Strong	Yes	Strong
Jarbin and Von Knorring (2004)	Moderate	Moderate	Strong	Moderate	Strong	Yes	Strong
Kirov et al. (1998)	Moderate	Moderate	Strong	Moderate	Strong	Yes	Strong
Mohr et al. (2006)	Moderate	Moderate	Weak	Strong	Strong	Yes	Moderate
Mohr et al. (2011)	Moderate	Moderate	Strong	Moderate	Strong	Yes	Strong
Mohr et al. (2012)	Strong	Moderate	Strong	Moderate	Strong	Yes	Strong
Nolan et al. (2012)	Moderate	Moderate	Strong	Strong	Moderate	Yes	Strong
Phillips and Stein (2007)	Moderate	Moderate	Strong	Strong	Strong	Yes	Strong
Röhricht et al. (2009)	Weak	Moderate	Strong	Moderate	Strong	Yes	Moderate
Rosmarin et al. (2013)	Moderate	Moderate	Strong	Moderate	Strong	Yes	Strong
Roystonn et al. (2021)	Moderate	Moderate	Strong	Strong	Moderate	Yes	Strong
Serfaty et al. (2020)	Moderate	Moderate	Moderate	Moderate	Moderate	Yes	Strong
Shah et al. (2011)	Moderate	Moderate	Strong	Strong	Strong	Yes	Strong
Siddle et al. (2002)	Moderate	Moderate	Strong	Moderate	Weak	Yes	Moderate
Siddle et al. (2004)	Moderate	Moderate	Weak	Strong	Strong	Yes	Moderate
Triveni et al. (2017)	Strong	Moderate	Moderate	Strong	Moderate	Yes	Strong

Abbreviation: EPHPP, Effective Public Health Practice Project (Thomas 2003).

**TABLE 2:** Quality assessment for qualitative studies—JBI tool.

Author (Year)	1. Is there congruity between the stated philosophical perspective and the research methodology?	2. Is there congruity between the research methodology and the research question or objectives?	3. Is there congruity between the research methodology and the methods used to collect data?	4. Is there congruity between the research methodology and the representation and analysis of data?	5. Is there congruity between the research methodology and the interpretation of the results?	8. Are participants, and their voices, adequately represented?	9. Is the research ethical according to current criteria or, for recent studies, and is there evidence of ethical approval by an appropriate body?	10. Do the conclusions drawn in the research report flow from the analysis, or interpretation, of the data?	Include if yes to 2–5, 8–10
Borras et al. (2007)	Yes	Yes	Yes	Yes	Yes	Yes	Yes	Yes	✓✓
Drinnan and Lavender (2006)	Yes	Yes	Yes	Yes	Yes	Yes	Unclear	Yes	✓
Hanevik et al. (2017)	Yes	Yes	Yes	Yes	Yes	Yes	Yes	Yes	✓✓
Heffernan et al. (2016)	Unclear	Yes	Yes	Yes	Yes	Yes	Yes	Yes	✓✓
Ho et al. (2016)	Yes	Yes	Yes	Yes	Yes	Yes	Yes	Yes	✓✓
Huguelet et al. (2007)	Yes	Yes	Yes	Yes	Yes	Yes	Yes	Yes	✓✓
Kirov et al. (1998)	Yes	Yes	Yes	Yes	Yes	Yes	Unclear	Yes	✓
Mohr et al. (2006)	Unclear	Yes	Yes	Yes	Yes	Yes	Yes	Yes	✓✓

Abbreviation: JBI, Joanna Briggs Institute Quality Assessment Tool (Lockwood et al. 2015).

## 3 Results

A total of 35 studies were included in this review from the initial search of 702 papers. The main findings of the eligible studies are summarised in Table 3.

**TABLE 3:** Characteristics of studies meeting inclusion criteria (*n* = 35).

Author (Year)	Country and type of study	Sample size and setting	Mean age (SD)	Questionnaire and diagnostic tools	Main findings and clinical implications
AbdelGawad et al., (2017)	Texas, USA; Cross sectional; Retrospective; Quantitative	Total Sample *n* = 175 93M/81F (data unavailable for one participant) Psychosis sample of interest: 92 (MDD with psychosis, BP with psychosis, SAD and SZ—no individual numbers specified) Inpatient	35 (11.5)	Duke University Religion Index (DUREL); Clinician Rated Dimensions of Psychosis Symptom Severity (CRDPSS).	Individuals with high scorers on the non‐organised religiosity scale were less likely to have psychosis than low scorers on the non‐organised religiosity scale (47% vs. 52%; *p* < 0.05). High non‐organised religiosity was significantly associated with increased psychosis severity compared with low non‐organised religiosity (14.5 ± 5 vs. 12.4 ± 6; *p* < 0.05). High non‐organised religiosity was significantly associated with more severe delusions compared with low non‐organised religiosity (2.82 ± 1.55 and 2.02 ± 1.82; *p* = 0.003), and high intrinsic religiosity was significantly associated with more severe auditory and visual hallucinations compared with low intrinsic religiosity score (2.07 ± 1.75 and 1.54 ± 1.70; *p* = 0.04). Organisational religiosity was not significantly related to psychosis severity. The high non‐organised religiosity group had a significantly longer length of hospital stay compared with the low non‐organised religiosity group (8.3 ± 3.8 vs. 6.9 ± 3.4 days; *p* < 0.05). Whereas high organisational religiosity and high intrinsic religiosity did not differ in length of hospital stay when compared with their respective low scores (however, these results are from the total sample, which includes individuals with mood disorders without psychosis; therefore, they may not relate solely to the psychosis population of interest). Results suggest both onset of psychosis, psychosis symptomatology and hospitalisation levels may vary depending on an individual's religious activity. Therefore, a brief measure of religious activities such as the DUREL may be able to identify individuals at a greater risk of psychosis.
Amadi et al. (2021)	Nigeria; Descriptive; Cross‐sectional; Quantitative	SZ: 156 73M/83F Outpatients	38.0 (12.2)	Duke University Religion Index (DUREL); The Positive and Negative Symptoms Scale (PANSS); Morisky Medication Adherence Scale (MMAS‐8).	No significant association was found between all measures of religiosity (organisational religious activity, non‐organisational religious activity, and intrinsic religious activity) and medication adherence. There was no significant association between organisational religious activity and various dimensions of psychopathology (PANSS positive, PANSS negative, PANSS Gen Psych). Non‐organisational religious activity and intrinsic religious activity had significant negative correlations with PANSS negative scores (*r* = −0.20, *p* < 0.01). Results display that religion does not play a significant role on medication adherence and that there was no significant association between organised religious activity and measures of psychopathology. However, non‐organised and intrinsic religiosity were associated with a reduction in the negative symptoms of psychosis. These results could imply that different domains of religiosity could have varying impacts on negative symptomatology and that we should address domains separately rather than looking at religiosity as one entity.
Anderson‐Schmidt et al. (2019)	Germany; Cross sectional; Quantitative	SZ: 215 SAD: 47 155M/107F Inpatients & outpatients	No religious delusions: 41 (13) Religious delusions: 40 (11) (No overall mean age specified)	Modified version of the Structured Clinical Interview for DSM‐IV Axis I Disorders (SCID‐I); Religious activity: self‐report questionnaire (created by the authors); Structured Clinical Interview for DSM‐IV‐TR Axis I Disorders (SCID‐1‐TR); Positive and Negative Symptoms Scale (PANSS).	The risk of experiencing religious delusions was significantly increased in patients with strong religious activity compared to patients without religious affiliation (OR = 3.6, *p* = 0.010). Low or moderate religious activity had no significant effect. Among the subgroup of patients with a lifetime occurrence of religious delusions, the acutely delusional patients did not score significantly higher on the self‐rating scale of religious activity compared those not acutely delusional. Results suggest that strong religious activity is a risk factor for the occurrence of religious delusions in SZ and SAD. Thus, clinicians may need to consider an individual's level of religiosity when approaching their care and consider whether religiosity is pathological. To identify pathological religiosity, it is important to investigate whether additional cognitive or behavioural abnormalities are present in an individual and whether there was a change in religious attitudes without external influences.
Borras et al. (2007)	Geneva, Switzerland; Cross sectional; Qualitative and Quantitative	Total Sample *n* = 103 SZ: 80% SAD: 18% Psychosis disorder not otherwise specified: 2% 70%M/30%F (Participant numbers not reported) Outpatients	38 (10)	Semi‐structured interview based on the Multidimensional Measurement of Religiousness/Spirituality for Use in Health Research (MMRS) the Religious Coping Index (RCI) and a questionnaire on spiritual and religious adjustment to life events; QOL was self‐rated by means of a visual analog scale; Positive and Negative Symptoms Scale (PANSS); Clinical Global Impression (CGI); DSM‐IV; Clinical interviews of patients and clinicians were used to assess adherence to treatment; Blood drug monitoring.	The researchers placed participants into three groups: Group 1: no religious affiliation/religion seen as unimportant; Group 2: presence of a religious affiliation/religion seen as important/no religious group practices; Group 3: presence of a religious affiliation/religion seen as important/religious practices in groups. Findings displayed that adherent patients belonged significantly more often to group 3 than nonadherent patients (*p* < 0.04). The more religion was important in patients' lives, the less patients were substance abusers (group 3: 10% vs. group 2: 30% vs. group 1: 47%*; x* ^2^ = 8.01, df = 2, *p* < 0.02) and the more they were in symptomatic remission (group 3: 39% vs. group 2: 12% vs. group 1: 27%*; x* ^2^ = 6.88, df = 2, *p* < 0.03). 31% of the participants stated that religion gave meaning to their illness; 26% stated that religion gave meaning to their illness with negative religious contents; and 43% adhered to the medical model of illness. In the group of medication adherent participants, medical representations of illness were more prominent than spiritual or religious representations; this was reversed for non‐adherent patients whereby religious representations of illness were prominent. Results display that religion and spirituality may be beneficial in preventing the maintenance of psychosis symptoms and promoting recovery. However, it is important to note that adherent participants adopted a medical representation of illness rather than a religious/spiritual. Thus, despite religious affiliation, medical representations of illness may improve recovery rates. These findings suggest that understanding an individual's religious affiliation should be considered in the clinical management of patients with psychosis and clinicians should aim to build a shared representation of illness with patients, where spirituality was found to be an important component of such representations.
Brito et al. (2021)	France; Cross sectional; Quantitative	Total Sample *n* = 38 694; Psychosis sample of interest: BP: 585 Psychosis symptoms: 9250 Psychosis disorders: 1019 Gender unclear Clinical setting unclear (French general population)	No mean age (age 18+)	Religiosity was assessed based upon religious beliefs and practice, identified by two questions: ‘are you a believer?’ and ‘are you religiously observant?’; Mini International Neuropsychiatric Interview (MINI).	Symptoms of psychosis were significantly more frequent among subjects with religious beliefs (OR = 1.37, *p* < 0.001) and with observance (OR = 1.14, *p* < 0.001). Regarding psychosis disorders, the associations were the same (beliefs: OR = 1.38, *p* < 0.001, and observance: OR = 1.25, *p* = 0.004). In contrast to the total sample, psychosis disorders and symptoms were not significantly associated with religious observance in the sample of believers. The results of this study concerning psychosis outcomes are in favour of a stronger association with religious beliefs rather than religious observance. These results are unique in that they consider different aspects of religiosity including beliefs and observance and it may be suggested that religious beliefs and observance should be considered when taking preliminary case histories. Future studies should aim to use more objective measures to understand whether religiosity is present in individuals before the onset of psychosis, as cross sectional studies currently do not infer causation, only a correlation.
Caqueo‐Urízar et al. (2015)	Bolivia, Chile, and Peru; Cross sectional; Quantitative	SZ: 253 164M/89F Outpatients	35.6 (15.5)	Survey for Relatives and Patients Regarding Causes of SZ; Drug Attitude Inventory (DAI‐10); Positive and Negative Symptoms Scale (PANSS).	Patients with higher magical‐religious causal beliefs had significantly lower DAI‐10 scores (*r* = −0.18, *p* < 0.01); and after adjustment for socio‐demographic and clinical factors, patients with higher magical‐religious causal beliefs had significantly lower DAI‐10 scores (*β* = −0.27, *p* < 0.05) and significantly more severe symptoms according to the PANSS positive factor (*β* = 0.17, *p* < 0.05). Results display that individuals holding magical‐religious beliefs experienced less favourable attitudes to medication and experienced more severe symptoms. Findings indicate that it could be beneficial for clinicians to address patients' causal beliefs as part of their treatment. However, results need to be replicated by prospective studies that can address the temporality of associations.
Caqueo‐Urizar et al. (2016)	Bolivia, Chile and Peru; Cross sectional; Quantitative	SZ: 253 66.4%M/33.6%F (no specific numbers provided). Outpatient	35.6 (15.5)	Religious involvement was assessed with a semi‐structured interview; Clinical Interview (spirituality and religiosity); SZ QOL Questionnaire (SQoL18); Positive and Negative Symptoms Scale (PANSS).	The S‐QoL 18 index was significantly and positively correlated with religious involvement (*p* = 0.002). Religious involvement was significantly associated with the S‐QoL 18 index (*b* = 0.13; *p* = 0.048) and with the autonomy dimension of the S‐QoL (*b* = 0.15; *p* = 0.027). Results display that religious involvement is positively related to QOL. However, this association is moderate and should be considered alongside other factors. These findings indicate that, if spirituality and religion is one way for patients to cope with their illness, other ways of coping should also be considered by clinicians.
Cohen et al. (2010)	New York City, USA; Cross sectional; Quantitative	Total sample *n* = 311 SZ sample of interest: 198 101M/97F Outpatients	61.5 (5.6)	Stress Process Model; The Religiousness Scale (developed for this study); Centre for Epidemiologic Studies Depression Scale (CES‐D); Positive and Negative Symptoms Scale (PANSS); Financial Strain Scale; Multi‐level Assessment Inventory; Instrumental Activities of Daily Living Scale (IADL); Cognitive Coping Scale; Lifetime Trauma and Victimisation Scale; Self Esteem Scale; Network Analysis Profile; Acute Stressors Scale; QOL Index (QLI).	Although there was an association between greater religiousness and lower PANSS positive scores, this did not attain significance. Religiousness had a significant, albeit modest, independent additive effect on QOL (*β* = 0.17, *tb* = 3.02, *p* = 0.003), however, it did not have any buffering effects on the four stressors that were significantly associated with QOL (financial strain, acute stressors, depression, and self‐esteem). After two other mediating variables (cognitive coping techniques and number of confidantes) were added, the association of the religiousness scale scores with the QLI scores remained significant. (*β* = 0.13, *tb* = 2.20, *p* = 0.024). Findings suggest that religiousness was not significantly associated with psychosis symptoms. However, religiousness may have a favourable impact on the QOL of older adults with SZ, and it must be considered along with other therapeutically important agents. Results shed light on the older adult population, which is often underreported in research. Additionally, these results stress that clinicians should not overlook patients' religious involvement, which may be a therapeutically important component.
Compton and Furman (2005)	Atlanta, USA; Cross sectional; Quantitative	SZ spectrum disorder: 18 (total sample) SZ: 12 Schizophreniform disorder: 5 SAD: 1 16M/2F Inpatient	22.3 (3.5)	The Spiritual Well‐Being Scale (SWBS); The Structured Clinical Interview for DSM‐IV Axis I Disorders (SCID‐I); Positive and Negative Symptoms Scale (PANSS).	PANSS positive symptom scores were not correlated with either religious or existential well‐being scores. PANSS negative symptom scores were significantly inversely correlated with religious well‐being scores (*ρ* = −0.614; *p =* 0.007), but not with existential well‐being scores. Results demonstrate that the PANSS negative symptoms were inversely associated with religious wellbeing, indicating that individuals with greater negative symptoms reported less wellbeing in terms of their relationship with God. However, as the religious wellbeing subscale of the SWBS refers to a personal belief in or relationship with God, it could be that those with greater negative symptoms correlated with deficits in affect or interpersonal relatedness, leading to lower levels of reported religious wellbeing. Further research is necessary to elucidate the extent and significance of these correlations. If more evidence follows this study's results, clinicians may need to consider addressing religious well‐being when addressing negative and general psychosis symptomatology.
Drinnan and Lavender (2006)	UK; Cross sectional; Qualitative	Diagnosed as experiencing delusions: 7 6M/1F Outpatients	30–53 (mean and SD not specified)	A semi‐structured interview was developed to investigate how participants' beliefs had evolved over time, and to explore how religion had been involved in this process.	Participants described positive aspects of religion, e.g., how religion could provide strength and a way of coping with the stresses of life, including mental‐health problems: ‘If I had no faith, I don't know how I'd get through it. No faith, no hope, no light at the end of the tunnel. I would end it’. Participants reported that religion could provide answers, highlighting that religion featured in their attempts to make sense of experiences, including psychological stress: ‘there were passages for each particular occasion or emotion or feeling, there was a particular place where you could find a solution in the Bible’. Some participants felt that religion could have a distressing effect on mental health: ‘You'll find most mental illness is caused by religious guilt’. Some individuals reported unusual religious experiences such as: being healed by god, casting evil out and conversing with the devil. Some individuals made religious attributions to explain their experiences: ‘I truly believe it was God, but I don't know why he wanted to confuse me’. Results display how religious background influences the nature of delusions and how these are attributed in both a positive and negative way. One of the most significant applications of this research is that religion may provide a framework to help make sense of what are often seen as confusing and distressing experiences. These interviews highlight that there is a lack of therapeutic space to discuss religious concerns. Therefore, it is important for clinicians to pay attention to religious beliefs and how this is incorporated into a client's background. Further research can build upon this work with a larger sample size and potentially incorporating quantitative methodology for more robust and generalisable results.
Esan and Lawal (2021)	Nigeria; Cross sectional; Quantitative	SZ: 215 109M/106F Outpatients	38.8 (9.4)	Daily Spiritual Experiences Scale (DSES); World Health Organisation Composite International Diagnostic Interview (WHO‐CIDI); Hamilton Depression Rating Scale (HDRS); Positive and Negative Symptoms Scale (PANSS); Social and Occupational Functioning Assessment Scale (SOFAS‐DSM‐IV); Remission in SZ Working Group (RSWG) criterion.	There was a significant relationship between spirituality and lifetime suicidal thoughts (*χ* ^2^ (1) = 4.7, *p =* 0.03); where participants who had low spirituality (33.3%) were more likely to have had suicidal thoughts in their lifetime than those with high spirituality (16.3%). The relationship between spirituality and remission was significant (*χ* ^2^ (1) = 14.61, *p* = < 0.001), where participants with high spirituality (85.7%) were more likely to be in remission than those with low spirituality (53.3%). Pearson correlation analysis indicated that spirituality was negatively correlated with the severity of negative symptoms (*r* = −0.24, *p* < 0.001), PANSS general symptoms score (*r* = −0.25, *p* < 0.001), total PANSS score (*r* = −0.26, *p* < 0.001) and the severity of depression (HDRS total score) (*r* = −0.33, *p* < 0.001); and positively correlated with functioning (SOFAS score) (*r* = 0.28, *p* < 0.001). Results display that participants with a high level of spirituality were less likely to have had suicidal thoughts, had less severe psychopathology, were more likely to be in remission and had better functioning than participants with a lower level of spirituality. These findings demonstrate that spirituality may be an inexpensive and effective adjunct to biomedical intervention in the management of patients with SZ and should therefore be better explored in the treatment of SZ. In future studies it may be beneficial to identify the direction of the association between spirituality and functioning and conduct studies that can separate the effects of religion from spirituality.
Fares et al. (2023)	Lebanon; Cross sectional; Quantitative	SZ: 125 SAD: 23 47M/101F Inpatients	Absence of religious hallucinations: 55.47 (12.98) Presence of religious hallucinations: 50.75 (13.88)	Scale for the Assessment of Positive Symptoms (SAPS); Positive and Negative Symptoms Scale (PANSS); Brief Religious Coping Scale (BRCOPE).	More religious negative coping (OR = 1.11, *p* < 0.05) was significantly associated with higher odds of having religious hallucinations. Results display that assessing the spirituality and religiosity in patients with SZ or SAD is an essential tool for understanding the patient as a whole. These results are important as misunderstanding the cultural background of patients with SZ may lead to poor communications and ethical dilemmas, inducing practise problems among mental health patients. Therefore, this study emphasises the importance of understanding religious and spiritual coping practises as negative religious coping may be predictive of developing religious themed hallucinations. Further research can be carried out with a larger sample size to clarify the association between religiosity and the development of religious hallucinations in a religiously diverse population.
Getz et al. (2001)	Ohio, USA; Cross sectional; Quantitative	SZ Spectrum Disorder: 49 SAD: 40 Affective Psychosis: 44 82M/51F Inpatients	Non religious: 31.2 (14.2) Protestant: 32.0 (7.8) Catholics: 33.3 (8.8)	Patients' religious background and their amount of religious activity prior to the index episode were obtained by the rater (ICC > 0.75 for religious activity score); Structured Clinical Interview for DSM‐IV Axis I Disorders Patient Version 2.0 (SCID‐I/P); Young Mania Rating Scale (YMRS); Hamilton Depression Rating Scale (HDRS); Scale for the Assessment of Positive Symptoms (SAPS).	When examining all of the patients, the amount of religious practice was correlated with higher ratings of religious delusions (*r* = 0.27, *p* < 0.01). The severity of religious delusions did not differ among religious groups in those patients who reported religious delusions. Results display that religious commitment is a clinically relevant phenomenon. The amount of religious activity prior to hospitalisation was a strong predictor of the ratings of religious delusions currently experienced, displaying an association between religious beliefs and delusions in patients with psychosis. These results suggest that religiosity can impact upon the experience of religious delusions, therefore, understanding an individual's religious affiliation may be essential during a clinician's assessment and formulation.
Hanevik et al. (2017)	Norway; Thematic analysis; Qualitative	FEP: 18 Gender not specified Sample drawn from the Early Treatment and Identification of Psychosis study (Norway).	Age not specified	Semi‐structured and in depth interview for religiousness.	11 out of 18 participants gave a religious explanation for their hallucinations. Participants described their religiousness to be helpful in coping with their disorder, giving meaning to life as well as a relationship to a sacred figure. However, religiousness often contained religious omnipotent delusions, and built on hallucinations. The misinterpretation of hallucinations as mystical experiences may reinforce individuals' delusional systems and prevent symptomatic recovery. Results suggest that the main characteristic of patients' religiousness is that they explain their experiences of hallucinations as mystical experiences, encompassing them into their belief systems. Findings display that there are both positive and negative connotations of religiousness when considering the maintenance and recovery of psychosis. These findings display the importance of taking patients' religiousness into account in psychotherapy. Additionally, clinicians holding knowledge on religion is important in assisting patients with finding a meaning in life and coping with psychosis.
Heffernan et al. (2016)	UK; Social constructionist grounded theory; Qualitative	Individuals with psychosis: 10 8M/2F Outpatient	25–35 (mean or SD not specified)	Religiosity was measured through a flexible, semi‐structured interview.	Several processes through which religion may influence recovery were identified: use of scriptures and rituals; a genuine connection with God; the struggle to maintain rituals; guidelines for living; choice and control; relating to others; enhancing psychological well‐being; and making sense of experiences. Use of scriptures and rituals: ‘the sooner I pray the sooner I get better’. A genuine connection with God: a reciprocal relationship with a higher power was considered to be a central factor in religion's role in recovery. Struggle to maintain rituals: the consequence of this took on a circular process whereby self‐blame and guilt were exacerbated. Guidelines for living: using guidelines helped individuals to navigate their difficulties and facilitate recovery. Others found that having strict rules made them unwell. Choice and control: participants stressed the importance of having control over their beliefs. Relating to others: religion was found to both enhance and hinder connections with others. Enhancing psychological wellbeing: through reducing shame in associated with difficult experiences, to fuel creativity, to enhance hope and to soothe emotions. Making sense of experiences: religion can assist individuals with reappraising experiences, but others found conflict between the psychological and medical models. Results display that religion can benefit recovery; however, it can also encourage self‐blame and guilt. These results suggest that it is essential for services to consider the religious needs of service users and reduce conflicting messages between mental health services and religious organisations. The results also indicate that spiritual wellbeing should continue following discharge and be available within community services. Future studies can adopt a wider and more diverse sample population to clarify results.
Ho et al. (2016)	Hong Kong; Qualitative with grounded theory	SZ (clients): 18 Mental health professionals (professionals): 19 Clients: 10M/8F Professionals: 8M/11F Outpatients	Clients: 28.4 (5.3) Professionals: 38.83 (6.2)	Spirituality was measured through a semi‐structured interview.	Clients and professionals regarded spirituality as an inherent part of an individual's wellbeing, clients' rehabilitation, and their lives in general. At the personal level, clients' descriptions were more factual, concrete, short term, and affective. On the other hand, professionals' descriptions were more abstract, complex, and cognitive. At the communal level, both client and professional parties had a similar understanding of spirituality but different interpretations of its role in recovery. The clients regarded spirituality as a source of giving and receiving love and care, whereas the professionals regarded it as a means of receiving support and managing symptoms. Findings highlighted similarities and differences between individuals with SZ and mental health professions in their views on spirituality and its impact on recovery. These findings suggest that a strength and ability focussed approach rather than a weakness and symptom focussed perspective on interventions may be beneficial for clients. Additionally, it may be feasible to conduct spiritual interventions for the SZ population as this may assist with fostering a stable mind and improve their sense of connectedness to the self and others.
Huang et al. (2011)	Taiwan; Cross sectional; Quantitative	SZ: 55 22M/33F Outpatients	37.2 (SD not specified)	A semi‐structured questionnaire collected information on: religious affiliation and religiosity; psychosis symptoms and religious delusion/hallucination; and treatment‐seeking behaviour, including experience of magico‐religious healing, satisfaction with psychiatric therapy, and treatment preference; Positive and Negative Symptoms Scale (PANSS); The Religiosity Measure self‐rating scale; Global Assessment of Functioning Scale (GAF); Measure of ‘satisfaction with psychiatric therapy’; The ‘preference of treatment’ scale.	Participants with religious delusions/hallucinations had significantly lower scores on functioning and significantly higher scores on religiosity than those without religious delusions or hallucinations and those not scoring highly on positive symptoms (*F* = 10.51, *p* < 0.05). Participants with religious delusions/hallucinations had significantly higher scores on religiosity compared to those without hallucinations/delusions (23.3 ± 7.1 vs. 17.0 ± 6.2). Higher scores of religiosity were found to significantly correlate with, longer duration of illness (*r* = 0.37, *p* < 0.05), lower preference of psychiatric treatment (*r* = −0.54, *p* < 0.05), lower functioning score (*r* = −0.39, *p* < 0.05), religious affiliation (*r* = 0.61, *p* < 0.05) and experiences of delusions/hallucinations (*r* = 0.30, *p* < 0.05). Patients with a religious affiliation showed significantly less preference towards psychiatric treatment than those without a religious affiliation (*t* = −4.25, *p* < 0.001), although they displayed similar levels of satisfaction with psychiatric treatment. Individuals with religious delusions/hallucinations were significantly more likely to receive magico‐religious healing (*X* ^2^ = 5.77, *p* = 0.035) and not be satisfied with treatment (*x* ^2^ = −2.30, *p* = 0.026) compared to those without religious delusions and hallucinations. Results demonstrate that religiosity/spirituality can impact religious delusions, duration of illness, functioning and preference of psychiatric treatment. Therefore, clinicians need to consider ways to encourage treatment seeking behaviour to help combat the presence of a potentially increased psychosis severity. Incorporating Socratic questioning into CBTp may encourage this.
Huguelet et al. (1997)	Switzerland; Longitudinal; Quantitative	SZ: 67 38M/29F Inpatients	Religiously practising participants: 27 (6) Non‐practising participants: 27 (7)	The researchers divided participants into two groups, (1) those taking part in regular activities with an established religion, or regular activities with a marginal religious group or sect; and (2) patients without religious activities (does not state which measure was used to ascertain this division); Disability Assessment Schedule (DAS‐II); Camberwell Family Interview (CFI); Axe V of the DSM‐IV; Global Assessment Scale (GAS); Criteria developed by Vaughn and Leff (1976) to measure relapse.	Although not significant there was a trend for practising patients to relapse less during the 5 year follow up when compared to non‐practising individuals. The difference in length of hospitalisation was not significant between the practising and non‐practising groups. Results display that religiously practising individuals tend to have fewer relapses as compared to the non‐practising group. However, the length of hospital stay was not significantly different. It could be probable that the patients who were less symptomatic and well adapted took part in religious movements, which maintained their social integration and subsequent health. Therefore, religion alone may not explain the outcome. These findings demonstrate that religious involvement in patients with SZ is frequent and tends to denote a subgroup with better outcomes, even if religion isn't the sole cause of this. Therefore, religion shouldn't be ignored in practise.
Huguelet et al. (2007)	Switzerland; Qualitative and quantitative; Cross sectional	SZ or SAD: 115 80M/35F Outpatient	Psychosis patients with suicide attempts: 39 (10) Psychosis patients without suicide attempts: 42 (10)	Religion and religious coping was assessed through a semi‐structured interview developed by the researchers; Subjective QOL was auto‐evaluated by means of a visual, analogical scale; Positive and Negative Symptoms Scale (PANSS); Clinical Global Impression (CGI); Psychosocial adaptation was evaluated with axis V of the DSM‐IV; Patients were asked about past suicide attempts and the number and methods used were reported.	Patients without suicidal attempts reported a protective role of religion more often than patients with suicidal attempts (31% vs. 20%), and less often an incentive role (3% vs. 17%) (*X* ^2^ = 7.63, df = 2, *p* < 0.02). For psychosis patients who had attempted suicide, the positive role of religion included religious coping, ethical condemnation of suicide and rediscovery of a meaning in life. For those who had not attempted suicide, religion played a protective role through fighting despair and restoring hope. For psychosis patients who had attempted suicide, negative aspects of religion were suicide attempts following a break with a religious community, attempts from religious delusions/hallucinations, a wish to die to be with God and a mystical experience of death. For those who had not attempted suicide, negative aspects of religion included a wish to die to be with God and anger with God. Results display that religion can be associated with suicide attempts; however, it can also act as a protective factor against suicide attempts. Interventions aiming to lower suicide rates in psychosis patients should take these results into account, considering the protective nature of religion and how this can be incorporated into care. However, it is important to note the two sided effects of religion on suicide and be cautious in addressing religion's potential facilitating role.
Huguelet et al. (2016)	Switzerland; Cross sectional; Quantitative	Total Sample *n* = 175 SZ sample of interest: 74 SZ sample of interest: 45M/29F Outpatients	SZ sample of interest: 45 (11)	Valued‐Living Questionnaire (VLQ) measuring the subjective importance of core domains in life (spirituality); Portrait Values Questionnaire (PVQ); Semi‐structured interview measuring religion and spirituality; Beck Depressive Inventory II (BDI II); Medication Adherence Rating Scale (MARS); Positive and Negative Symptoms Scale (PANSS); Social Functioning Questionnaire (QFS); Barrat Impulsivity Scale for Impulsivity (BIS); Beck Hopeless Scale; State–Trait Anger Expression Inventory (STAXI); Rosenberg Self‐esteem Scale (RSE); World Health Organisation QOL BREF (WHOQoL‐BREF); Revised Life Regard Index (LRI‐R).	For individuals with SZ whom spirituality is essential demonstrated significantly better social functioning (*x* ^2^ = −3.58, *p* < 0.001), fewer negative symptoms (*x* ^2^ = −2.17, *p* < 0.05), and a higher level of self‐esteem (*x* ^2^ = −2.74, *p* < 0.01); and felt that their own lives featured a better psychological (*x* ^2^ = −2.22, *p* < 0.05) and social (*x* ^2^ = −2.17, *p* < 0.05) quality compared to those who did not see spirituality as essential. Results display that essential spirituality was associated with an improved clinical state (social functioning, negative symptoms) and better mental health (self‐esteem, psychological and social QOL) in those with SZ. These results suggest that religiosity and spirituality should be considered in recovery‐oriented care.
Jarbin and Von Knorring (2004)	Sweden; Explorative study; Quantitative	SZ spectrum disorder: 45 (SZ: 34; SAD: 10; and schizophreniform disorder:1) Affective psychosis disorder: 37 (BP: 25; MDD with psychosis features: 12). Psychosis disorder due to a medical condition: 1 Psychosis disorder due to substance abuse: 3 Psychosis disorder not otherwise specified: 2 Gender not specified Inpatients	15.79 (1.5)	The Swedish Register of Death was contacted in all cases of death at follow‐up; Questions regarding suicide attempts were asked at every follow‐up interview with patients, parents/caregivers, and information on suicide attempts was searched for in psychiatric registers and case records; Positive and Negative Symptoms Scale (PANSS); Brief Psychiatric Rating Scale (BPRS); Strauss‐Carpenter employment sub‐scale and social contacts subscale; Global Assessment of Functioning Scale (GAF); Lancashire QOL Profile (LQOLP); Follow up information was mainly obtained through: personal interviews, interviews with parent or case manager, psychiatric records from adult psychiatric services and with records from child psychiatry only.	Patients with no suicide attempt and psychosis disorders reported a better subjective QOL in the domains religious belief compared to those who had attempted suicide (*p* = 0.005). Satisfaction with religious belief was the only QOL domain still correlated (*r* = −0.30) to suicide attempts after controlling for concurrent symptoms of anxiety and depression. The association remained on the verge of significance after controlling for both anxiety and depression and nicotine dependence. Findings suggest that satisfaction with religious belief could have an independent inverse correlation to suicide attempts. However, it may be more important to consider factors such as symptoms of anxiety and depression. These results demonstrate that religiosity may have a positive impact in preventing suicidal behaviour, therefore, should be considered in recovery oriented care.
Kirov et al. (1998)	UK; Cross sectional; Qualitative and quantitative	SZ: 30 BP: 15 Schizophreniform disorder: 5 SAD: 2 26M/26F Inpatients in hospital	35.2 (11.1)	Semi‐structured interview regarding religious beliefs and activities; Schedule for Assessment of Insight; Compliance with medication was assessed on a 7‐point scale ranging from complete refusal to active participation.	65.2% of participants explained that their religious faith had not changed after the onset of illness; in only two cases there was a reduction in the strength of the faith; and in 12 patients (26.1%) there was an increase in the religious faith after the onset of illness. Patients who used religion for coping with their illness were more likely to be compliant with medication, although this relationship did not obtain significance. Results display that religious faith often remains during psychosis or becomes stronger during illness. Additionally, religion may play a positive role in medication compliance. Clinicians can be reassured by these findings, knowing that deeply religious individuals can have good insight into their illness and continue to comply with medication. However, it is important that clinicians can identify individuals who rely heavily on religious coping as they can then direct resources appropriately. Future research should attempt to explore the prospective changes in religious beliefs related to changes in mental state, and whether such beliefs and coping strategies have a beneficial effect on psychosis.
Mohr et al. (2006)	Geneva, Switzerland; Cross sectional; Qualitative and quantitative	SZ: 92 SAD: 21 Psychosis disorder not otherwise specified: 2 80M/35F Outpatients	39 (10)	Positive and Negative Symptoms Scale (PANSS); Clinical Global Impression (CGI); Psychosocial adaptation was evaluated with axis V of DSM‐IV. The authors developed a semi‐structured interview to measure religiosity/spirituality; A visual analog scale with 5 anchored points to obtain self‐report measures on the salience of religiousness, religious coping, and synergy with psychiatric care was used; The subjective importance of religion for the patient was also assessed by the two clinicians, in terms of its centrality.	Patients were separated into three groups reflecting their coping strategies at a psychological level: positive religious coping, negative religious coping and no religious coping. Positive religious coping gave participants a positive sense of self, in terms of hope, meaning of life, enjoyment of life and self‐respect; and a meaning to their illness, mainly through positive religious connotations and less frequently with negative connotations. Even if meanings were negative in religious terms, they were positive in psychological terms by fostering an acceptance of the illness. Negative religious coping was found to cause despair and suffering. On clinical variables, the no religious coping group was significantly more likely than the other two groups to be regular substance users (47% vs. 19*%; X* ^2^ = 6.1*8*, df = 2, *p* < 0.05) and had significantly more negative symptoms (median score = 16 vs. 10; *H* = 7.9*2*, df = *2, p* < 0.02). Duration of illness was significantly greater in patients with negative religious coping (median = 17 years vs. 12 years; *H* = 8.2*4*, df = *2, p* < 0.02), and patients in this group were not as happy as the others (20% vs. 47%; *H* = 5.9*2*, df = *2, p* < 0.05). Results suggest that positive religious coping has more favourable outcomes than negative religious coping; and, in some instances, using positive or negative religious coping may be more beneficial than not using religious coping. These results suggest that religion should be assessed within each patient and that collaboration with religious individuals may be a unique way to support psychosis patients where religion is of importance.
Mohr et al. (2011)	Switzerland; Longitudinal; 3 year follow up from Mohr et al. (2006); Quantitative	SZ and SAD: 92 (no diagnostic specific data provided). Demographic information only provided for those with positive and negative religious coping: 89 58M/31F Outpatients	Positive coping: 38.89 (10.52) Negative coping: 42.28 (6.72)	Structured interview for religiosity and spirituality; A visual analog scale with 5 anchored points to obtain self‐report measures on the salience of religiousness, religious coping, and synergy with psychiatric care was used; Positive and Negative Symptoms Scale (PANSS); Clinical Global Impression (CGI); Global Assessment of Functioning Scale (GAF); The patients estimated their QOL with a Visual Analogue Scale ranging from 0 (very unhappy) to 10 (very happy); Mini International Neuropsychiatric Interview (MINI).	Compared to individuals with negative religious coping, subjects with positive religious coping expressed significantly more often a ‘happy’ subjective QOL (*N* = 32 (42%) vs. *N* = 2 (15%), *b* = 2.22, *p =* 0.023). For individuals with positive religious coping, ‘subjective importance of religion to cope with symptoms’ was associated with positive changes in global evaluation of clinical and functional status (*X* ^2^ = 10.35; df = 4; *p* = 0.035). Results display that positive religious coping can positively impact QOL and functioning. This longitudinal study gives more weight to the possibility of a causal role of religion in the outcome of SZ. Positive religious coping may alter the course of psychosis by promoting psychological recovery through coping strategies. Therefore, these results could have an indirect but important implication for clinical practise.
Mohr et al. (2012)	Switzerland, Canada and North Carolina; Quantitative; Cross sectional	SZ: 208 SAD: 68 164M/41F Outpatients	45 (13)	Positive and Negative Symptoms Scale (PANSS); Clinical Global Impression (CGI); Global Assessment of Functioning Scale (GAF); Semi‐structured interview on the role of spirituality and religiousness in participants' lives and to cope with their illness; Social Functioning Questionnaire (QFS); Index of Self‐Esteem Rating Scale (SERS); Medication Adherence Rating Scale (MARS); World Health Organisation QOL BREF (WHOQoL‐BREF); Huber Centrality Scale.	Participants reporting negative religious coping experienced significantly more antagonism with psychiatric treatment (Factor 2: m = 0.54 +/ 1.48 Vs. m = −0.08 +/ 0.88; *t* = 3.55, df = 274, *p* < 0.01), were less involved and supported by their religious communities (Factor 3: m = −0.38 +/ 0.76 vs. m = 0.06 +/ 1.02; *t* = −2.45, df = 274, *p* < 0.01), were significantly more severely ill (*t* = 3.35, df = 273, *p* = 0.001; *t* = 2.94, df = 273, *p* = 0.004), presented with significantly more positive symptoms (*t* = 4.28, df = 273, *p* = 0.000), reported significantly worse social functioning (*t* = 3.51, df = 273, *p* = 0.001), were less adherent with their medication (although not significant), and reported a significantly poorer QOL (*t* = 3.02, df = 273, *p* = 0.003) compared to participants with positive religious coping. Results display the largely detrimental effect negative religious coping has on individuals with psychosis. It may be beneficial for clinicians to consider this in care, discouraging the use of negative religious coping and encouraging the use of positive religious coping.
Nolan et al. (2012)	Southeastern USA; Cross sectional; Quantitative	SZ: 46 SAD: 17 33M/30F Outpatients	42.2 (11.6)	Semi‐structured, in person, audiotaped interviews and scales; World Health Organisation QOL BREF (WHOQOL‐BREF); Brief Religious Coping Scale (BRCOPE).	Positive religious coping was significantly correlated to the QOL facet of psychological health (*r* = 0.28, *p* = 0.03). Negative religious coping and QOL were significantly inversely related (*r* = −0.30, *p* = 0.02). Positive religious coping was significantly associated with psychological health in the reduced univariate general linear model (*B* = 0.72, *p* = 0.03, *R* ^2^ = 0.08). Results highlight the importance of religion and spirituality in coping with mental illness, and suggest that greater awareness of the importance of religion in this population may improve cultural competence in treatment and community support. If these findings are validated, then greater awareness of the importance of religion in this population may lead to improved cultural competency in treatment, services, and community support and strengthen collaboration between clinical and religious community‐based organisations to improve social integration.
Phillips and Stein (2007)	Ohio, USA; Longitudinal; Quantitative	SZ: 22 BP: 26 (43 of the 48 participants completed interviews at both time points). 26M/22F Outpatients	24 (3.4)	Three meaning making subscales of the Religious Coping Scale (RCOPE); The Brief Symptom Inventory (BSI); The Global Symptom Index (GSI); Personal Loss from Mental Illness Scale (PLMI); Stress‐Related Growth Scale (SRGS); The Scales of Psychological Well‐Being (PWB); Semi‐structured interviews.	At Time 1, stronger beliefs that mental illness was an opportunity to grow spiritually (BRR) was correlated with significantly higher levels of reported psychological growth due to mental health problems (*r* = 0.30, *p* < 0.05). Similarly, Time 2 BRR scores were significantly and positively correlated with Time 2 stress‐related growth scores (*r* = 0.48, *p* < 0.05) and levels of self‐reported psychological well‐being (*r* = 0.32, *p* < 0.05). Individuals viewing their mental illness as a punishment from God (PGR) at Time 1 reported significantly lower levels of psychological well‐being (*r* = −0.46, *p* < 0.05), higher levels of personal loss (*r* = 0.43, *p* < 0.05), and greater psychological distress (*r* = 0.43, *p* < 0.05). Time 2 PGR was correlated with significantly higher levels of Time 2 loss (*r* = 0.52, *p* < 0.05) and greater psychological distress (*r* = 0.59, *p* < 0.05). Findings display that the use of benevolent religious reappraisals had positive implications for individuals coping with serious mental illness, while greater reliance on punishing God reappraisals had more negative mental health implications. Study findings can serve to remind practitioners that young adults diagnosed with SZ or BP can use religious forms of coping to make meaning of their experiences. Results can increase practitioners' sensitivity to both helpful and unhelpful roles that religion can play when coping with serious mental illness.
Röhricht et al. (2009)	UK; Exploratory Cross‐sectional study; Quantitative	Acute paranoid SZ: 42 Gender unclear Inpatients	White Caucasian: 36.8 (8.9) Black Caribbean: 39.9 (13.9) Black African: 39.4 (9.6) Asian: 29.4 (7.1) (Total mean and SD not provided).	Structured Clinical Interview for DSM‐IV Mental Disorders (SCID); Positive and Negative Symptoms Scale (PANSS); Ego Psychopathological Inventory (EPP); The Manchester Short Assessment of QOL (MANSA); The Spiritual Transcendence Scale (STS); The Religious Orientation Test (ROT); A semi structured, qualitative interview to measure religiosity.	For the Spiritual Transcendence Scale, a significant and positive association with the degree of ego‐vitality scores (*F* = 6.1, *p* = 0.017, *r* = −0.34) was displayed. For the Religious Orientation Test a significant and positive association with the degree of PANSS‐ negative scores (*F* = 4.4, *p* < 0.05, *r* = 0.39) was displayed. This indicates a lower degree of spirituality and religiosity for those patients with higher psychopathology scores and vice versa. The QOL (MANSA) scores did not correlate with measures of spirituality and religiosity. These results suggest that religiosity exerts a positive effect on health, and demonstrate that clinicians need to be more mindful to fully address religious, spiritual and cultural factors in individual patients, and incorporating knowledge from such research findings may assist in increasing meaningful clinical competencies.
Rosmarin et al. (2013)	Massachusetts, USA; Prospective study; Quantitative	Current psychotic disorder: 23 Past psychosis disorder: 24 (i.e., SZ, SAD, or mood disorder with psychotic features—no individual diagnostic numbers specified). 42.5%M/57.5%F (no participant numbers reported) Outpatients.	29.72 (10.62)	Mini International Neuropsychiatric Interview (MINI); Global Assessment of Functioning Scale (GAF); Interview prior to treatment to assess for suicidality over the past month; Self‐report/interview measures of religious involvement and religious coping; Self‐report measures assessed: depression, anxiety, psychological well‐being and psychosis; Religious affiliation was assessed with three items: What is your religious preference?; To what extent do you believe in God?; and How important is religion in your life?; Two items from the Duke University Religion Index (DUREL) to assess for public/private religious activity; Brief Religious Coping Scale (BRCOPE); Suicidality Module from the Miniature International Neuropsychiatric Interview (MINI); 24‐item Behaviour and Symptom Identification Scale (BASIS‐24); Centre for Epidemiologic Studies Depression Scale (CES‐D); Penn State Worry Questionnaire; The Schwartz Outcome Scale.	Negative religious coping was associated with substantially higher suicidality, accounting for 46.24% of the variance in frequency of ideation (*r* = 0.68, *p* < 0.001), and 37.2% of the variance in intensity of ideation (*r* = 0.61, *p* < 0.001). Negative religious coping was also associated with greater depression (*r* = 0.41, *p* = 0.006), a trend towards greater anxiety (*r* = 0.33, *p* = 0.10), less psychological well‐being (*r* = −0.41, *p* = 0.02), and psychosis symptoms (*r* = 0.22, *p* ≤ 0.01), prior to treatment. Over the course of treatment, positive religious coping was associated with significantly greater improvements in depression (*r* = 0.50, *p* = 0.004), anxiety (*r* = 0.60, *p* < −0.001) and psychological wellbeing (*r* = −0.37, *p* ≤ 0.05). Results display that negative religious coping was generally associated with a worse outcome within the sample, whereas, positive religious coping was associated with substantially greater treatment gains in depression, anxiety and psychological wellbeing. The strength of these relationships suggests that spiritual struggle potentially represents a significant safety concern for psychosis patients. Therefore, this domain warrants specific assessment as a potential risk factor in psychiatric care. In light of the associations between spiritual struggle and distress observed, it is possible that the use of positive, adaptive and functional religious belief and practice as a coping strategy is particularly helpful for individuals with psychosis.
Roystonn et al. (2021)	Singapore; Cross sectional; Quantitative	Individuals with psychosis: 364 (no specific diagnosis specified) 168M/196F Outpatients	35.2 (10.8)	World Health Organisation QOL BREF (WHOQOL‐BREF); Brief Religious Coping Scale (BRCOPE); Duke University Religion Index (DUREL); Covariates used in this study included sociodemographic, health, and clinical factors, known to independently affect QOL outcomes in persons with psychosis.	Positive religious coping was significantly associated with better scores on physical (*β* = 0.51, *p* = 0.02) and psychological (*β* = 0.64, *p* = 0.01) QOL domains. Negative religious coping was related to significantly worse QOL in all four domains: physical (*β* = −0.44, *p* = 0.03), psychological (*β* = −0.76, *p* < 0.01), social (*β* = −0.54, *p* = 0.03), and environment (*β* = −0.65, *p* < 0.01). Increased participation in organisational religious activities was significantly and positively associated with higher QOL for psychological (*β* = 2.47, *p* < 0.01), social relationships (*β* = 2.66, *p* = 0.01), and environment (*β* = 2.09, *p* = 0.01) domains. Those with no religious affiliation were found to have significantly higher scores in the QOL domain for social relationships (*β* = 4.5*9, p* = 0.02). Overall, these findings suggest that both positive and negative religious coping are associated with QOL in outpatients living with psychosis and, religion may have a significant influence on the individual's wellbeing and recovery from illness. These findings demonstrate that spiritual care should be incorporated in treatment plans to benefit recovery, reduce relapse, and improve the QOL of mental health patients.
Serfaty et al. (2020)	Israel; Cross sectional; Quantitative	SZ: 19 SAD: 10 Postpartum psychosis: 1 15M/15F Inpatient and outpatient	36.0 (12.1)	Clinical Global Impression (CGI); Brief Psychiatric Rating Scale (BPRS); Two items were chosen to assess general religiosity based on Koenig's (2018) review of basic religious variables: (1) belief in God and (2) importance of religion; Brief Trust/Mistrust in God Scale; Jewish Religious Coping Scale (JCOPE); QOL Enjoyment and Satisfaction Questionnaire—Short Form; Treatment credibility/expectancy was assessed using two of the 6‐item treatment credibility and expectancy questionnaire. The measure is divided into treatment credibility and treatment expectancy; Suicidality module from the Miniature International Neuropsychiatric Interview (MINI).	Trust and mistrust in God were significantly and positively correlated with treatment expectancy (*r* = 0.44, df = 28, *p* = 0.015; *r* = 0.43, df = 28, *p* = 0.019, respectively). Among men, negative religious coping on the JCOPE was significantly correlated with lower treatment credibility (*r* = −0.52, df = 13, *p* = 0.045). However, all other correlations were not statistically significant. Among women, positive religious coping was significantly correlated with higher treatment expectancy (*r* = 0.52, df = 13, *p* = 0.045) and with higher QOL (*r* = 0.52, df = 13, *p* = 0.045); and Trust in God was correlated with significantly lower BPRS total score (*r* = −0.56, df = 13, *p* = 0.03) and with higher treatment expectancy (*r* = 0.67, df = 13, *p* < 0.01). Results display that adaptive religious coping serves as a protective factor and a positive indicator of treatment expectancy. The link of negative religious coping to reduced treatment credibility among men may reflect the tendency of men to engage in more avoidance that translates into greater resistance to treatment or scepticism of treatment. This research has important implications as the authors bring attention to an underrepresented clinically relevant phenomenon in an underrepresented sample. Additionally, due to stigma and other cultural barriers the Jewish population experience, religious factors may be crucial in building motivation and encouraging adherence to medication.
Shah et al. (2011)	India; Cross sectional; Quantitative	Residual SZ: 103 64M/39F Outpatient	34 (8.3)	WHO QOL‐Spirituality, Religiousness and Personal Beliefs Scale (WHOQOL‐SRPB); Positive and Negative Symptoms Scale (PANSS).	The domain of Spiritual, Religiousness and Personal Beliefs and all its facets other than spiritual connection correlated significantly (*p* < 0.001) and positively with all other domains of QOL and overall QOL. Results display that the spirituality and religiosity domains of QOL have an important influence on many aspects of QOL of patients with SZ. Besides pharmacological and non‐pharmacological management for SZ, clinicians should assess the spirituality/religiosity status and its meaning to an individual patient and should encourage those patients to turn to religion more frequently if they consider it useful in dealing with their suffering.
Siddle et al. (2002)	UK; Cross sectional; Quantitative	SZ: 145 SAD: 31 Schizophreniform disorder: 15 Delusional disorder: 1 (one participant diagnosis not stated out of 193 total participants) 135M/58F Inpatient	35.1 (SD not specified)	An algorithm for reliably establishing religious delusions was developed; Positive and Negative Symptoms Scale (PANSS); Global Assessment of Functioning Scale (GAF); The Hallucination Scale and the Delusion Scale from PSYRATS; Religiosity was assessed using the patients' own classification; The presence of religious delusions was established using questions from the Present State Examination (PSE).	The religiously deluded subjects were found to be significantly more religious on self‐assessed degree of religiosity than were the comparison group (*U* = 2433.5, *p* = 0.015). Being religious is significantly more likely to be associated with religious delusions (*x* ^2^ = 4.18 [1], *p* = 0.041). The odds of an individual self‐identified as religious also having religious delusions is more than twice that of someone who does not identify as religious (OR = 2.29). The correlation coefficient between self‐assessed religiosity and the PANSS positive scale was neither strong nor statistically significant. Results display that individuals with religious delusions tended to score more highly than the other patients with SZ who did not have religious delusions, on measures of religiosity. Additionally, findings suggest that individuals with religious delusions may have a greater need for religion at times of crises, therefore, clinicians should be promoting this more in clinical care.
Siddle et al. (2004)	UK; Prospective cohort; Naturalistic; Longitudinal; Quasi‐experimental; Quantitative	Total Sample (who could be re‐assessed at follow up) *n* = 155 SZ: 116 SAD, schizophreniform disorder and delusional disorder were the diagnoses held by the remaining participants‐ no numbers stated. 108M/47F Inpatient	36 (SD not specified).	The authors used Siddle et al. (2002) methodology for understanding whether participants had religious delusions or not (see above); Positive and Negative Symptoms Scale (PANSS); Self‐report measures were used to separate the religious from non‐religious patients.	There were no significant differences between the religious and non‐religious patients on psychopathology at the initial interview. The difference between the religious and non‐religious patients after treatment was also not significant. Results suggest that religiosity does not impact psychopathology or treatment response in individuals with psychosis. Future studies should extend the follow up time beyond four weeks to ascertain any differences in outcome which can impact upon clinical implications.
Triveni et al. (2017)	India; Cross sectional; Quantitative	Total sample *n* = 150 SZ sample of interest: 100 56M/44F Outpatients	35.6 (10.8)	Religiousness Measure Scale; Duke University Religion Index (DUREL); Brief Religious Coping Scale (BRCOPE); Positive and Negative Symptoms Scale (PANSS); Global Assessment of Functioning Scale (GAF); World Health Organisation QOL BREF (WHOQOL‐BREF) (Hindi version).	When considering the correlations between PANSS scores and measures of religiosity (religious involvement, religious influence, religious hope, total religiosity, religious attendance, private religious activity, intrinsic religiosity, positive RCOPE and Negative RCOPE), PANSS‐negative symptom score, PANSS general psychopathology symptom score, and total PANSS score, significantly and negatively correlated with various aspects of religiosity (*p* < 0.05). There were few significant negative correlations between PANSS‐positive symptom subscale score and various domains of religious measure scale (*p* < 0.05). There were significant positive correlations between the GAF score and various aspects of religiosity (*p* = < 0.05) except for negative RCOPE score. There were significant positive correlations between QOL and various aspects of religiosity (*p* = <; 0.05) except for negative RCOPE score. Results display that higher religious involvement and practices are associated with lower levels of negative, general, and total PANSS scores. With regard to positive symptoms, the relationship was not as consistent and as strong as seen for other symptoms. Overall religious involvement and practices were associated with a better level of functioning in the present study; and religious involvement, religious practices, and use of religious coping are associated with better QOL. Thus, encouraging individuals to participate in religious activities can reduce levels of residual psychopathology and improve patients QOL. There is a need for clinicians to change their approach, whereby they should enquire about patients' religiosity and spirituality. They should also encourage patients to use positive religious coping strategies.

Abbreviations: BP, bipolar; CBTp, cognitive behavioural therapy for psychosis; DSM‐IV, diagnostic and statistical manual of mental disorders, fourth edition; F, female; FEP, first episode psychosis; M, male; MDD, major depressive disorder; QOL, quality of life; SAD, schizoaffective disorder; SZ, schizophrenia.

### 3.1 Maintenance

#### 3.1.1 Symptomatology

Symptoms of psychosis were significantly more frequent among individuals with religious beliefs and observance (observing or participating in religious group activity, Burazeri et al. 2008) than among individuals without religious beliefs and observance (OR = 1.37 and OR = 1.14, respectively) (Brito et al. 2021). Additionally, religiosity and holding magical‐religious causal beliefs (religious/spiritual explanations for the occurrence of psychosis, Patel 1995) were significantly and positively associated with positive symptoms of psychosis (Caqueo‐Urízar et al. 2015; Huang et al. 2011). Conversely, religiosity was significantly and negatively associated with negative symptoms (Amadi et al. 2021; Compton and Furman 2005; Esan and Lawal 2021; Huguelet et al. 2016; Röhricht et al. 2009; Triveni et al. 2017), general symptoms (deficits in cognition e.g., disorientation, poor attention and social avoidance, Shankar and Nate 2007), and total symptoms (the sum of positive, negative and general symptoms) (Esan and Lawal 2021; Triveni et al. 2017). Alternatively, four studies reported no significant correlation between religiosity/spirituality and positive symptoms (Cohen et al. 2010; Compton and Furman 2005; Siddle et al. 2002) and total symptoms (Siddle et al. 2004). The discrepancy in findings might be explained by the employment of varying religious measures across studies. The non‐significant findings were derived from semi‐structured interviews to assess subjective religiosity or employed a religiousness scale designed specifically for their study, while the significant findings employed validated measures of religiosity such as the Duke University Religion Index. Therefore, the body of findings appears to implicate the role of religiosity/spirituality in the maintenance of positive symptoms of psychosis.

Studies investigating individuals' religious practices found that high non‐organised religiosity (NOR) (individually conducted religious activities, Francis et al. 2019) was significantly associated with more severe delusions compared with low NOR (mean 2.82 vs. 2.02, respectively); and high intrinsic religiosity (IR) (the degree of personal religious commitment, Francis et al. 2019) was significantly associated with more severe auditory and visual hallucinations compared with low IR (mean 2.07 vs. 1.54, respectively) (AbdelGawad et al. 2017). However, no significant correlations were found between organisational religiosity (communal religious activities e.g., attending public places of worship, Francis et al. 2019) and psychosis symptoms (AbdelGawad et al. 2017; Amadi et al. 2021). These results suggest that practising religion on an individual basis may increase the severity of positive symptoms in comparison to practising at a group or community level.

#### 3.1.2 Religious Delusions

Patients with strong religious activity had a significantly higher risk of experiencing religious delusions compared to those without religious affiliation (OR = 3.6, *p* = 0.01 vs. OR = 1.2, *p* = 0.68, respectively); low to moderate religious activity had no significant effect (Anderson‐Schmidt et al. 2019). Additionally, the amount of religious practice significantly and positively correlated with religious delusions (Getz et al. 2001; Huang et al. 2011; Siddle et al. 2002), which arose from their hallucinatory experiences (Hanevik et al. 2017). However, for individuals with a lifetime occurrence of religious delusions, religious activity did not differ between acutely and non‐acutely delusional individuals (Anderson‐Schmidt et al. 2019).

#### 3.1.3 Religious Coping

Individuals often used religious coping to manage psychosis experiences and to provide meaning in life (Drinnan and Lavender 2006; Hanevik et al. 2017; Mohr et al. 2006). Positive Religious Coping (PRC) (e.g., prayer, meditation, community support, religious affirmation) was found to significantly improve individuals' wellbeing (Rosmarin et al. 2013), QoL (Mohr et al. 2011; Nolan et al. 2012; Roystonn et al. 2021; Serfaty et al. 2020), treatment expectancy (Serfaty et al. 2020) and psychological health (Nolan et al. 2012) including depression, anxiety (Rosmarin et al. 2013) and clinical and functional status (Mohr et al. 2011). Additionally, PRC was associated with increased medication adherence, although this finding was statistically non‐significant, which may be attributed to the study's small sample size (*n* = 30) rendering it underpowered to detect statistically significant between‐group effects (Kirov et al. 1998). These results suggest that PRC appears to improve individuals' mood, functioning, and treatment adherence during psychosis.

Alternatively, Negative Religious Coping (NRC) (e.g., only seeking Traditional Faith Healers, avoiding treatment, exorcism practices, self‐condemnation) was found to significantly increase positive symptoms of psychosis (Fares et al. 2023; Mohr et al. 2012), total psychosis symptoms (Rosmarin et al. 2013), illness duration (Mohr et al. 2006), antagonism with treatment (Mohr et al. 2012), suicidality (Rosmarin et al. 2013), depression (Rosmarin et al. 2013) and illness severity (Mohr et al. 2012); and significantly reduced QoL (Mohr et al. 2012; Nolan et al. 2012; Roystonn et al. 2021), happiness (Mohr et al. 2006), treatment credibility (Mohr et al. 2012; Serfaty et al. 2020), psychological wellbeing (Rosmarin et al. 2013) and social functioning (Mohr et al. 2012). NRC was also correlated with reduced medication adherence (Mohr et al. 2012) and a trend towards greater anxiety (Rosmarin et al. 2013). These results suggest that NRC has adverse effects on psychosis symptomatology, treatment response, mood, and functioning. Compared to individuals with PRC or NRC, individuals who did not use any religious coping were significantly more likely to misuse substances and had significantly more negative symptoms (Mohr et al. 2006). These findings suggest that PRC was associated with positive clinical outcomes, whereas NRC and the absence of any religious coping strategies were associated with negative clinical outcomes.

#### 3.1.4 Treatment Adherence & Attitudes

Medication adherent individuals were significantly more likely to be religious and to practice in groups compared to religious individuals with no group practices or those with no religious affiliation (Borras et al. 2007). Additionally, a trend towards significance (possibly owing to the study being underpowered) was found for the association between positive religious coping and medication compliance (Kirov et al. 1998). These results suggest that being religious/spiritual may encourage medication adherence. However, one study reported a statistically non‐significant association between religiosity (organisational religiosity, NOR, and IR) and medication compliance (Amadi et al. 2021). This discrepancy in findings might be attributed to the differing ways in which medication adherence was assessed across these studies. For example, the latter finding employed a self‐report measure (Morisky Medication Adherence Scale), which may be subject to social desirability bias, while the trend towards significance finding measured medication adherence via a clinician rated 7‐point scale ranging from observing client refusal to full compliance with treatment, which may be deemed a more objective measure of medication adherence. Additionally, medication adherent individuals tended to endorse a bio‐psychosocial explanatory model rather than a religious/spiritual explanatory model for their psychosis, while the opposite pattern was observed for non‐adherent individuals (Borras et al. 2007). These results suggest that holding a religious/spiritual conceptualisation can negatively impact an individual's adherence to medication.

With regards to treatment expectancy and attitudes, trust in God and PRC were significantly and positively correlated with treatment expectancy (Serfaty et al. 2020). However, NRC (Serfaty et al. 2020), holding magical‐religious causal beliefs (Caqueo‐Urízar et al. 2015), and higher scores of religiosity (Huang et al. 2011), were significantly and negatively correlated with treatment expectancy. These results suggest that, although PRC can be beneficial, higher levels of overall religiosity and holding magical‐religious causal beliefs can negatively impact attitudes to treatment.

#### 3.1.5 Duration of Illness & Hospitalisation

Religiosity was significantly and positively correlated with illness duration (Huang et al. 2011). However, the difference in length of hospitalisation was not significant between practising and non‐practising individuals (Huguelet et al. 1997). These findings highlight that religiosity/spirituality may increase the total duration of illness, but not length of hospitalisation.

### 3.2 Recovery

#### 3.2.1 Remission

Individuals who considered religion important in their lives and those with high levels of spirituality were both significantly more likely to be in remission (Borras et al. 2007; Esan and Lawal 2021). Both clients and professionals consider spirituality integral to rehabilitation (Ho et al. 2016), with clients believing that religion aids recovery through scriptures and rituals, offering life guidelines, and helping to make sense of their experiences (Heffernan et al. 2016). However, clients noted that religion could hinder recovery by causing self‐blame from unmet rituals, imposing lifestyle guidelines, and creating conflict with psychological and medical conceptualisations of illness (Heffernan et al. 2016). These findings suggest that religiosity/spirituality can increase the chances of remission. However, qualitative studies indicate that opinions vary on whether it helps or hinders, highlighting that religion is an individualistic phenomenon.

#### 3.2.2 Relapse

One study found a non‐significant trend toward lower relapse rates in religiously practising patients over a 5‐year period (Huguelet et al. 1997). However, more research is needed to clarify this relationship.

#### 3.2.3 Suicide

For individuals who had attempted suicide, negative aspects of religion included separation from their religious community, a desire to be with God, and a mystical experience of death (Huguelet et al. 2007). These results suggest that religiosity/spirituality might influence suicidal behaviour. However, some individuals who had attempted suicide recalled positive aspects of religion, such as religious coping, ethical condemnation of suicide, and rediscovering meaning in one's life (Huguelet et al. 2007). Thus, while conflict with one's faith may have precipitated suicide attempts for some, it was a source of prevention for others.

Individuals who had never attempted suicide reported a significantly better QoL in the domain of religious belief compared to those who had attempted suicide (Jarbin and Von Knorring 2004) and reported a protective role of religion through fighting despair and restoring hope (Huguelet et al. 2007). However, despite having no suicide attempts, some individuals reported negative aspects of religion, such as anger with God or a desire to be with God after death (Huguelet et al. 2007). Therefore, while religion may prevent suicide attempts for some, it may be a source of distress for others.

Regarding suicidal ideation, individuals with low levels of spirituality were significantly more likely to have experienced suicidal thoughts at some point in their lives (Esan and Lawal 2021). Moreover, NRC was significantly and positively correlated with the frequency and intensity of suicidal ideation (Rosmarin et al. 2013). Therefore, these results suggest that religiosity/spirituality can prevent suicidal ideation; however, individuals should avoid NRC mechanisms within religious practices.

#### 3.2.4 Quality of Life, Mood, & Functioning

Spirituality/religiosity was positively correlated with QoL in eight out of nine studies (Caqueo‐Urizar et al. 2016; Cohen et al. 2010; Huguelet et al. 2016; Mohr et al. 2011; Roystonn et al. 2021; Serfaty et al. 2020; Shah et al. 2011; Triveni et al. 2017). Religiosity/spirituality was significantly and positively associated with improved functioning (Esan and Lawal 2021; Triveni et al. 2017), psychological growth, stress‐related growth, psychological wellbeing (Phillips and Stein 2007) and ego‐vitality (Röhricht et al. 2009); and was significantly and negatively correlated with depression (Esan and Lawal 2021). Additionally, individuals who viewed spirituality as essential, demonstrated significantly higher social functioning and increased self‐esteem (Huguelet et al. 2016). Furthermore, appraisals of psychosis as a punishment from God was significantly correlated with decreased well‐being and significantly higher levels of distress and personal loss (Phillips and Stein 2007). Therefore, an individual's appraisals of one's psychosis experience appears to determine one's mood and functioning.

## 4 Discussion

Spirituality/religiosity was found to play a significant role in the maintenance and recovery of psychosis. Regarding the maintenance of psychosis symptoms, spirituality/religiosity was positively correlated with positive symptoms of psychosis and the latter was significantly more frequent among individuals with religious beliefs and observance than among individuals without religious beliefs and observance. Additionally, individualised religious practice was significantly correlated with more severe delusions compared with group religious practice. Moreover, high IR was significantly correlated with more severe auditory and visual hallucinations compared with low IR. However, no significant correlations were found between communal religious activities and psychosis symptoms. These results suggest that practising religion on an individual basis may increase positive symptom severity in comparison to practising at a group or community level. The body of findings therefore appears to implicate the role of religiosity/spirituality in the maintenance of positive symptoms of psychosis and the role of individualised religious practice in the severity of delusions and hallucinations.

According to the Cognitive Model of Psychosis (Garety et al. 2001), social isolation, as exemplified by individualised religious practice, contributes to the acceptance of incorrect appraisals of psychosis, an externalising attributional style, and poor social understanding (e.g., ‘God is punishing me’) by reducing access to alternative, normalising explanations. Alternatively, group religious practices may prevent the emergence of positive psychosis symptoms by providing social support, the sharing of beliefs, and the opportunity for reality testing through others' experiences.

Increased religious activity was associated with a significantly higher risk of religious delusions compared to those without religious affiliation. It has been suggested that delusional ideas lie on a continuum with normal beliefs (Harrow et al. 1988) and that religiously delusional individuals may have shifted along the continuum from normal religiosity to overvalued ideas, and subsequently to religious delusions (Siddle et al. 2002). Therefore, with increased religiosity, individuals may ascribe psychosis symptoms to a spiritual/religious conceptualisation, thereby manifesting more solidified religious delusions. These findings are consistent with the Cultural Formulation for Delusions and Hallucinations (Ghanem et al. 2023), which posits that anomalous experiences in psychosis symptom emergence may reflect pre‐existing cultural, religious, and spiritual themes held by the individual. This model proposes that cultural, religious, and spiritual factors influence one's perceptions, as well as personal, familial, and societal expectations regarding one's conduct/behaviour. Therefore, the violation of cultural/religious norms or the discrepancy between current circumstances and idealised cultural expectations may induce stress in the individual, giving rise to the formation of anomalous experiences such as hallucinations and delusions. The content of the latter may therefore reflect spiritual themes such as punishment from God, black magic, witchcraft or Jinns. One's appraisal of emerging symptoms and ensuing model of understanding (Religious/Spiritual vs. Bewitchment vs. Bio‐psychosocial) will therefore determine distress level and subsequent help‐seeking behaviours (Ghanem et al. 2023).

In terms of coping, PRC was found to improve wellbeing, QoL, and treatment expectancy, while NRC increased the severity of positive symptoms, illness duration, and suicidality, and reduced social functioning. Individuals without religious coping were more likely than those with PRC or NRC to abuse substances and exhibited more negative symptoms. This may be due to the sense of community and purpose provided by religiosity, which helps prevent social isolation and alternative coping mechanisms, such as substance misuse. Indeed, social isolation from religious groups has been found to increase the risk of engaging in substance misuse (Chou et al. 2011; Day and Rosenthal 2019). Conversely, holding a religious affiliation and attending religious events has been found to reduce substance misuse and loneliness (Ford and Hill 2012; Gallucci et al. 2018; Lauder et al. 2006). Therefore, PRC was associated with positive clinical outcomes, whereas NRC and the absence of any religious coping were associated with negative clinical outcomes.

Furthermore, group religious practice was significantly associated with increased medication adherence. Indeed, organisational religiosity significantly increased perceived social support and medication adherence across diagnoses (Hatah et al. 2015; Lin et al. 2018; Wahab et al. 2021). Despite this, heightened religiosity negatively correlated with treatment expectancy. This may be due to individuals' initial reluctance to take medication due to alternative coping mechanisms. However, once prescribed, individuals may recognise the benefits of medication and may draw on religious communities to support their adherence. Medication adherent individuals and those with a higher treatment expectancy tended to endorse a bio‐psychosocial explanatory model rather than a religious/spiritual explanatory model for their psychosis. The bio‐psychosocial explanatory model of psychosis has been positively associated with insight (Saravanan et al. 2007), while religious/spiritual explanatory models have been associated with an increased likelihood of visiting traditional faith healers (Chadda et al. 2001; Saravanan et al. 2007), poorer service engagement, and a longer duration of untreated psychosis (DUP) (Ghanem et al. 2023).

Religiosity was found to positively correlate with illness duration. However, the difference in length of hospitalisation was not significant between practising and non‐practising individuals. This may in part be explained by the need to promptly treat via medications and to plan discharges once a period of stabilisation has been reached. However, religion may increase illness duration as alternative coping mechanisms, such as NRC, may be more freely utilised outside the hospital setting, thereby preventing recovery.

In terms of recovery, religiosity/spirituality was found to improve remission rates (Esan and Lawal 2021). However, some individuals perceived religion to hinder recovery (e.g., by opposing bio‐psychosocial explanatory models). Alignment between clients' and clinicians' explanatory models has been proposed as integral to treatment and patient satisfaction (Callan and Littlewood 1998; Kleinman 1980). Indeed, individuals who attributed psychosis to spiritual causes or sought treatment from traditional faith healers had a longer DUP (Burns et al. 2010; Mbewe et al. 2006). Therefore, when individuals hold spiritual/religious models of illness and clinicians hold opposing explanatory models, recovery may be delayed. This therefore highlights the need for clinicians to effectively engage clients through the demonstration of curiosity of religious/spiritual themes and the use of socratic questioning to explicate their models of understanding and religious/spiritual appraisals (see Tables 4 and 5). Only after a comprehensive assessment of the client's spirituality, explanatory models, appraisals of psychosis symptoms, and validation of distress can the bio‐psychosocial model then be proposed as an alternative explanatory framework for their unusual experiences. The clinician can then subsequently explore with the client whether this alternative bio‐psychosocial conceptualisation (e.g., I experienced too much stress leading me to develop psychosis) is more helpful and less distressing than their religious/spiritual conceptualisation (e.g., Hearing the voice of God as punishment for my sins/feeling that one's body is possessed by demons [Primary appraisal], which means that I am bad [Secondary appraisal]). If the client is able to consider the possibility of having developed psychosis and views it as less distressing than their initial spiritual/religious conceptualisation, then the onset of psychosis can be normalised, bio‐psychosocial relapse prevention work can commence, and the importance of engaging with evidence‐based treatments for psychosis can be outlined.

**TABLE 4:** Identifying adaptive, grandiose and negative appraisals for anomalous experiences.

Adaptive appraisals
This might be a symptom of psychosis rather than a spiritual experience
My intense spiritual beliefs/psychosis might have been a response to extreme stress
This might be a spiritual test of my faith
I have developed psychosis but find prayer and the bible comforting
I was seeking spirituality but might have become overwhelmed by it leading to psychosis
Grandiose appraisals
I am enlightened and have a special mission from God
I have reached a high state of spiritual enlightenment
I am the chosen one
I am the Messiah
I have healing powers
Negative appraisals
I believe I am being punished for my sins
This burning sensation is a sign that I am going to hell
Seeing red eyes in others means that they are demons
I need to experience this physical pain in my body before I can receive redemption

**TABLE 5:** Socratic Questions to explore spirituality and religiosity in psychosis.

Engagement
I'm not an expert in religion but we can have a think together about what is causing you distress/what led you to come to our service, how does that sound?
Do you follow a particular faith/view yourself as spiritual?
What faith were you brought up with?/Do you still follow it?/has your faith changed at all over time?
How do you practice your faith/spirituality, what do you tend to do?
Is there anything you find helpful/unhelpful about it?
You mentioned that you are a spiritual person, is it possible that God/the universe is guiding you to our service to help you to get better?
Elucidating client's explanatory model (Religious/spiritual vs. bio‐psychosocial)
What's your understanding of what led you to our service? Do you think it was mental health related/something called ‘psychosis’ or a religious/spiritual experience you went through?
It sounds like it was an intense experience for you, which you believe was spiritual. How did your family/friends view this experience you went through? Did they also think it was something spiritual or something else?
Did you experience any stress in the lead up to coming to our service? What was happening in the 1–2 months before you came to our service/had your hospital admission? [explore bio‐psychosocial stressors]
Exploring the Meaning of having had a religious/spiritual experience versus a psychosis episode
What would it mean to you if you had an intense spiritual experience? [E.g., I am special/chosen/bad].
What would it mean to you if you did experience something called ‘psychosis’? [E.g., that the bullies won and I became unwell/I can't cope with stress].
What's the worst thing about that for you? [Downward arrow technique to elicit core beliefs such as weak/vulnerable, failure, defective, inferior, bad, unlovable/unlikeable, worthless].
Is that something you could accept, that you might have experienced too much stress, leading to some unusual/intense experiences but you can get better with medication and psychology so we can prevent this from happening again?
Exploring religious coping
What are you seeking when you search for religious videos online/passages from the bible?
You mentioned reading the bible [religious texts] after your psychosis, have any passages helped at all? Which ones? And what did you like about them?
Is there someone in your religious community (e.g., priest) you can ask these specific questions to, to see if your religious beliefs fit with their religious teachings?
You mentioned spending hours watching religious videos, what effect does this have on you? [distressed/preoccupied]
Can you do anything else instead? [talk to family member/listen to music/go for a walk]
Do you feel a particular emotion in the lead up to you searching for these religious videos/passages? [e.g., guilt]
What happens just before you feel this intense emotion? [emotional triggers]
Does anything help with lessening the intensity of this emotion for you?
Exploring psychosis appraisals
What makes you think that you are going to hell?
Is there anything you have done that makes you convinced that you are going to hell?
I can imagine this isn't an easy thing to discuss but you mentioned having some blasphemous thoughts, could you say a bit more about this? When did it start? What was happening at the time?
How do you know you are the chosen one/messiah?
You mentioned having special powers/healing abilities, when did you discover this and what were you able to do?
Interventive questioning
We all make mistakes, what about the idea of self‐forgiveness? Prayer? Trying to grow from our errors?
It sounds like you enjoyed some aspects of what you went through (e.g., feeling invincible and powerful) but were there any negative aspects to this experience? [arguments with family/employers, resulted in police being called/hospitalisation]
Regardless of whether it was a spiritual experience or not, can we have a think about managing stress to try and keep you out of hospital/prevent family from becoming worried about you?
Can I provide you with some information on biological, psychological, and social factors that can cause stress and you can let me know if any of it fits with what you went through in the lead up to your intense spiritual experience/hospital admission?

Conversely, if the experience of psychosis is seen as more distressing than their religious/spiritual explanation, then identifying their specific appraisals regarding having developed psychosis is necessary. For example, if it was not a religious/spiritual experience (e.g., possession) then it means that I developed psychosis/could not cope with stress (Primary appraisal). Using the CBT downward arrow technique regarding what that would say about them as a person would elucidate secondary appraisals in the form of core beliefs (e.g., then it would mean that I am weak/vulnerable, defective, a failure, bad, worthless, unlikeable/unlovable, and/or inferior). These core beliefs could then be targets for intervention by testing the evidence for and against these beliefs, as well as how helpful they are to hold, coupled with enhancing more positive beliefs about the self (e.g., I am strong/worthwhile/can grow from this experience).

Alternatively, if the religious/spiritual conceptualisation pertained to a grandiose appraisal concerning being chosen by God/having healing powers (Primary appraisal), it is likely to lead to secondary appraisals such as ‘I am special/superior to others’ and associated affect such as grandiosity and elation. Exploring what it would mean to the client if they were mistaken, namely that their intense religious/spiritual experience may be better explained by having developed psychosis, may give rise to feelings of disappointment for not having been ‘chosen’ coupled with the need to take medication for their psychosis. Indeed, if the client firmly holds on to grandiose appraisals (I am the Messiah/chosen one/have healing powers/have reached spiritual enlightenment) over having developed psychosis, then the clinician can explore the extent of life disruption caused by this intense spiritual experience (e.g., people did not understand me, family were worried, was approaching strangers to heal them, police were called, led to my hospital admission). Acknowledgement of the disruption caused by this experience, regardless of whether it was a spiritual experience or not (i.e., psychosis), can then allow for conversations about what may have precipitated this event by identifying bio‐psychosocial stressors in the lead up to this intense religious/spiritual experience and what may help to prevent it from occurring again (i.e., learning ways to manage stress, improve sleep, take medication). Therefore, identifying primary and secondary appraisals regarding religious/spiritual experiences vs. having developed psychosis would elucidate clinically meaningful intervention targets. It also highlights that clinicians and clients can still engage in meaningful work despite holding differing explanatory models.

In terms of religiosity and suicide, the findings are mixed, with some individuals viewing religion as a contributing factor to suicide and others viewing it as a deterrent. Possible mediating factors proposed for this discrepancy include social support, acceptance, and religious coping (Moreira‐Almeida et al. 2006). Additionally, an individual's locus of control (LOC) may explain idiosyncratic interpretations of the effect of religion on suicide. An internal LOC tends to be associated with wellbeing, while an external LOC tends to be associated with depression and anxiety. Religious beliefs tend to favour an internal LOC, which then positively impacts one's mental health (Levin and Schiller 1987; Moreira‐Almeida et al. 2006; Pargament et al. 1988).

Spirituality/religiosity was found to positively correlate with QoL, functioning, and wellbeing, and negatively correlated with depression (Esan and Lawal 2021). However, appraisals of psychosis as a punishment from God was associated with decreased wellbeing and higher levels of distress, highlighting that appraisals influence the relationship between psychosis symptoms and mood/functioning.

### 4.1 Religious Coping Model for Psychosis

**FIGURE 2:**
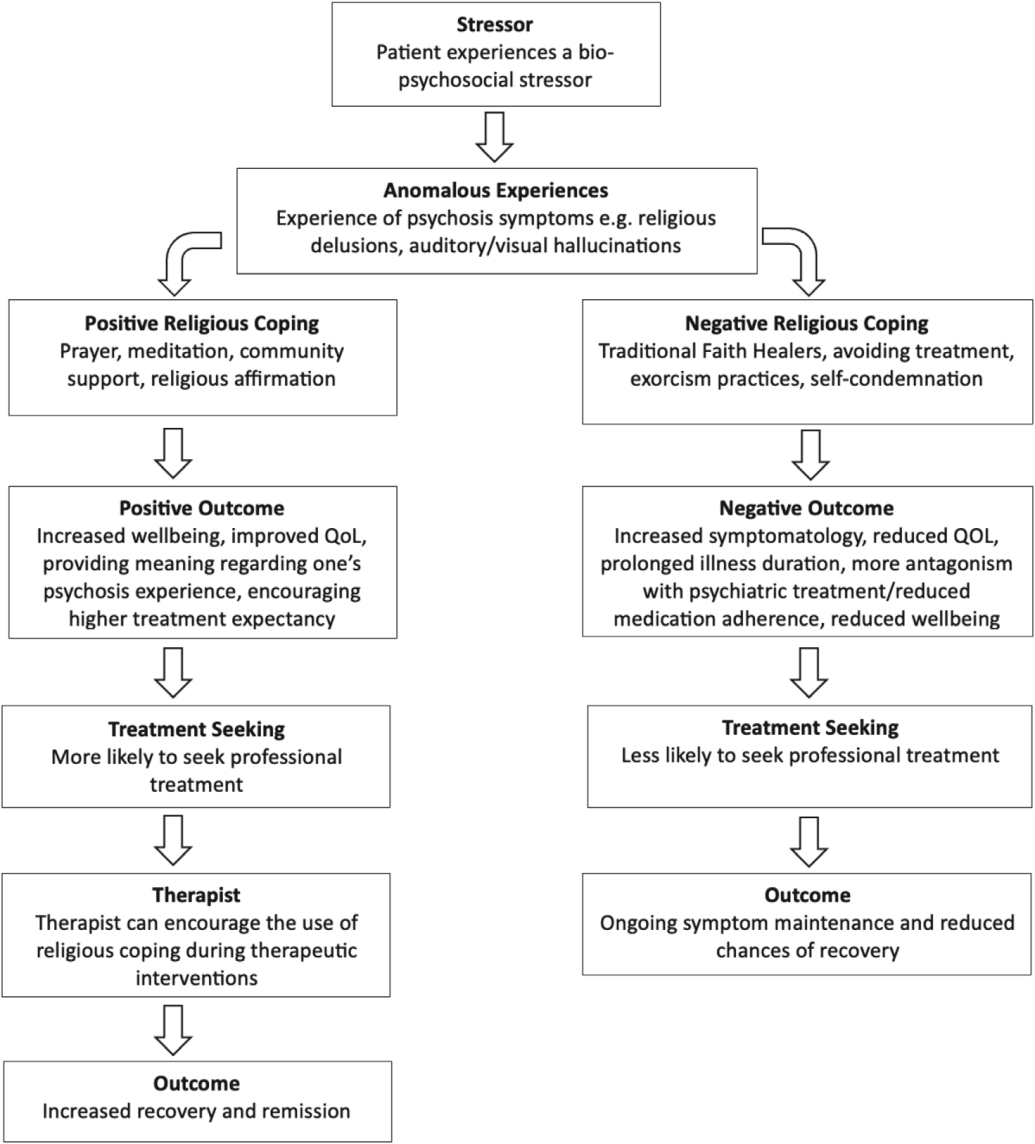
Religious Coping Model for Psychosis‐A conceptual framework for elucidating the outcomes of positive and negative religious coping in psychosis.

Based on the findings of the present review, we propose the following *Religious Coping Model for Psychosis* (see Figure 2), namely that the type of religious coping employed in response to anomalous experiences generated by bio‐psychosocial stressors influences clinical outcome. Negative religious coping entails only seeking traditional faith healers and exorcism practices, which would delay the initiation of evidence‐based treatments for psychosis. This in turn would increase symptomatology and reduce quality of life. Alternatively, positive coping encompasses prayer and endorsing religious affirmations, which may provide a sense of comfort, wellbeing, and meaning regarding one's psychosis experience. This in turn would increase help‐seeking behaviours, engagement with services, and adherence to treatment. Clinicians can therefore encourage the ongoing use of positive religious coping strategies presented by clients in order to foster recovery and remission, while respectfully discouraging negative religious coping strategies in favour of evidence‐based treatments for psychosis.

### 4.2 Cognitive Model of Spirituality and Religiosity in Psychosis

**FIGURE 3:**
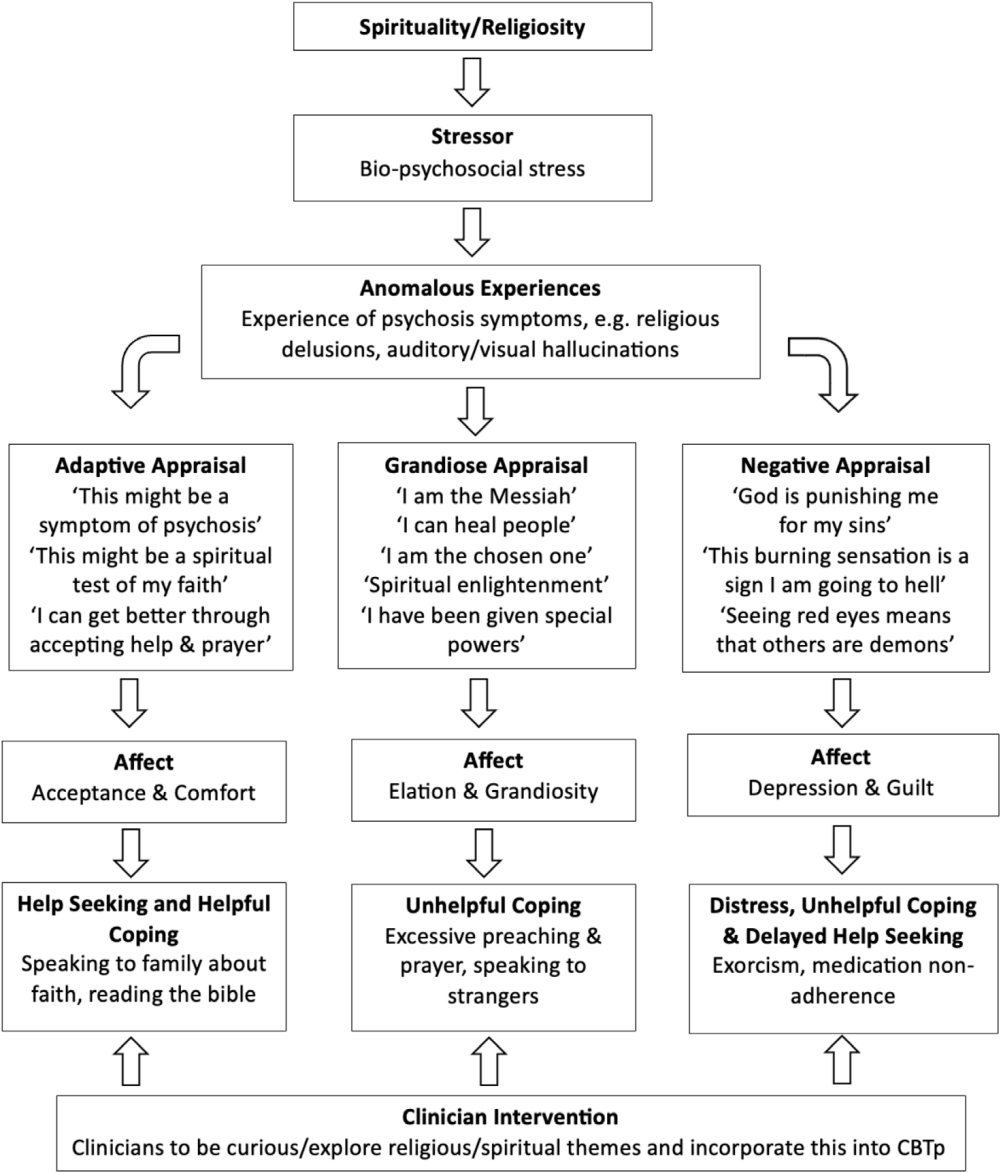
Cognitive Model of Spirituality and Religiosity in Psychosis‐A conceptual framework for identifying spiritual/religious themes in psychosis, subsequent appraisals, affect and coping.

Given the prominence of spiritual and religious themes in psychosis, we propose the following *Cognitive Model of Spirituality and Religiosity in Psychosis* (see Figure 3). This model advocates that spirituality and religiosity may serve as a precursor to idiosyncratic beliefs about oneself, others, and the world. Bio‐psychosocial stressors contribute to the generation of anomalous experiences such as hallucinations and delusions, which are then appraised according to three possible pathways, adaptive, grandiose, or negative (see Table 4). Each of these appraisals in turn contributes to an affective state and a subsequent coping behaviour. For example, adaptive appraisals (e.g., this might be a symptom of psychosis) are followed by an affective state typically characterised by acceptance and comfort, which leads to help‐seeking behaviours and helpful coping responses, such as treatment adherence and prayer. Clinicians can encourage these adaptive coping responses alongside medical and therapeutic interventions. Alternatively, grandiose appraisals (e.g., I am the chosen one) are followed by an affective state characterised by elation and grandiosity, which leads to unhelpful coping responses such as excessive preaching and disinhibited behaviours. Moreover, negative appraisals (e.g., God is punishing me for my sins) are followed by an affective state characterised by depression and guilt, which leads to distress, unhelpful coping responses, and delayed help‐seeking. Elucidating the appraisal of the anomalous experience (e.g., seeing auras means that I am the chosen one), along with the associated affect (e.g., elation), would then allow for socratic questions (see Table 5) and the exploration of more adaptive ways of thinking and coping. The proposed cognitive formulation model seeks to enhance engagement by working with clients' model of understanding, which would then enable the development of personalised and idiosyncratic formulations by incorporating religiosity and spirituality within CBTp practices.

## 5 Strengths and Limitations

This systematic review sought to summarise the role of spirituality/religiosity in the maintenance and recovery of psychosis. Employing a mixed methods synthesis of quantitative and qualitative studies enabled the assimilation of the available literature regarding the role of spirituality/religiosity in psychosis, which is a strength of the current review (Sandelowski et al. 2006). For example, combining the subjective experiences of religiosity/spirituality from the qualitative investigations along with the quantitative data of religiosity measures has enabled a comparison of both sets of findings. From these findings, this review has proposed two novel cognitive models, namely the *Religious Coping Model for Psychosis* (see Figure 2) and the *Cognitive Model of Spirituality and Religiosity in Psychosis* (see Figure 3), both of which can be used to enhance culturally adapted CBTp practices.

In terms of limitations, there is a risk of not capturing relevant papers from the search outputs. Additionally, the exclusion of unpublished literature may have limited the synthesis of the present review. As the majority of studies were cross‐sectional, it allowed for correlational analyses to be explored but precluded causal inferences from being made. Moreover, there was great variability in the measures of religiosity employed across studies, ranging from semi‐structured interviews, algorithms and scales created specifically for studies, as well as the use of validated measures. This would increase the heterogeneity of participant responses regarding definitions/concepts of spirituality/religiosity, rendering comparisons across studies difficult. Future studies would benefit from employing validated measures of spirituality/religiosity to enable comparisons across studies. Lastly, it was observed that the majority of studies included in the present review were conducted in Western countries (Europe, USA, Israel) (*n* = 25) compared to non‐western countries (*n* = 10), with the fewest studies conducted in the Middle East (*n* = 1), Africa (*n* = 2), South America (*n* = 2), and Asia (*n* = 5). This is a limitation regarding the evidence to date, owing to the limited perspectives from highly populous non‐western countries. This therefore highlights the need for future studies from non‐western nations to examine the role of spirituality and religiosity in psychosis.

### 5.1 Clinical Implications

Spirituality/religiosity has been found to significantly influence the maintenance and recovery of psychosis. Clinical services to date have tended to neglect this aspect of patients' lives possibly owing to the understandable prioritisation of treatment administration and management of safety. However, the exploration of one's spirituality/religiosity within clinician assessments and its relation to symptoms of psychosis may yield fruitful information regarding the development of personalised formulations, which would inform targeted interventions in CBTp. Moreover, if themes of spirituality/religiosity remain unaddressed in care, it could hinder engagement with services/treatment‐seeking, leading to ongoing symptom maintenance, resulting in delayed recovery. To overcome this, clinicians would benefit from assessing and understanding clients' explanatory models, as the latter can influence how psychosis symptoms are appraised and coped with. Indeed, through the employment of socratic questioning of spiritual/religious themes (see Table 5), clinicians can collaboratively devise a symptom‐specific spiritual formulation with clients employing the *Cognitive Model of Spirituality and Religiosity in Psychosis* (see Figure 3). This model proposes that individuals' appraisals (see Table 4) of anomalous experiences give rise to affective responses and subsequent coping behaviours; therefore, elucidating appraisals, affect, and coping would then allow for the exploration of more adaptive ways of thinking and coping. As such, we have also proposed the *Religious Coping Model for Psychosis* (see Figure 2), which demonstrates adaptive and maladaptive religious coping methods and how they respectively influence clinical outcomes. Therefore, clinicians could foster the use of PRC to aid in recovery and provide psychoeducation regarding the bio‐psychosocial model, emphasising the importance of engaging in evidence‐based treatments for psychosis, as opposed to potentially harmful religious practices such as exorcisms in the first instance.

As clinicians have tended to overlook spirituality/religion in care (Abdulla et al. 2019; Goldfarb et al. 1996), cultural competence training informed by religious community leaders may help clinicians to better understand clients' psychosis attributions. Additionally, involving religious leaders and religious organisations in encouraging help‐seeking and medication adherence would aid in reducing DUP and improved engagement with clinical services, thereby increasing chances of recovery and remission. Additionally, employing culturally adapted CBTp (CaCBTp), which pertains to adjusting the delivery of CBTp to different cultures and religions, could also foster engagement with services (Naeem 2012).

### 5.2 Future Directions

Future research could helpfully investigate the role of spirituality/religiosity across the psychosis spectrum (e.g., Clinical High Risk, First Episode Psychosis and Schizophrenia) and across stages of the illness (onset, maintenance, and recovery), as this would elucidate the role religiosity/spirituality might play throughout the course of illness. Additionally, the employment of longitudinal designs to observe how religious practices and beliefs effect the onset and development of psychosis over time would be clinically meaningful. Furthermore, as there are limited findings regarding the relationship between spirituality/religiosity on psychosis emergence, more studies are needed to explore this relationship. Indeed, the exploration of mediating factors for the relationship between spirituality/religiosity and psychosis symptoms, relapse, remission, and suicidality would help elucidate possible protective vs. risk factors. Possible mediating factors might include the role of emotions, appraisals, and meta‐cognitive beliefs (e.g., assigning heightened significance to anomalous experiences via a spiritual lens). Finally, more qualitative investigations would elucidate the subjective experiences regarding the role of religiosity/spirituality in the onset, maintenance, and recovery of psychosis, as well as identifying specific appraisals, emotions, and coping behaviours associated with religious delusions.

## 6 Conclusion

Spirituality/religiosity was found to play a significant role in the maintenance and recovery of psychosis. In terms of maintenance, religiosity/spirituality was positively correlated with positive symptoms of psychosis, with individualised religious practice being significantly associated with increased delusional severity compared with group religious practice, and high intrinsic religiosity being significantly associated with increased severity of auditory and visual hallucinations compared with low intrinsic religiosity. In terms of recovery, PRC was found to improve wellbeing, QoL, treatment expectancy, and medication adherence, while NRC increased suicidality, positive symptom severity, and illness duration, and reduced social functioning. Holding religious/spiritual explanatory models was associated with increased psychosis symptom severity and delayed recovery, while holding a bio‐psychosocial explanatory model assisted with recovery. The *Religious Coping Model for Psychosis* can aid clinicians in collaboratively exploring helpful and unhelpful religious coping practices with the view to reducing unhelpful coping and increasing positive coping methods. The *Cognitive Model of Spirituality and Religiosity in Psychosis* would enable clinicians to develop personalised, idiosyncratic, and symptom‐specific religious/spiritual formulations, elucidating appraisals and behaviours, which could be intervention targets within CBTp. Therefore, incorporating spirituality/religiosity within CBTp assessment, formulation, and interventions would facilitate culturally adapted practices, which would enhance engagement and foster recovery from psychosis among different cultural groups.

## Data Availability

Data sharing not applicable to this article as no datasets were generated or analysed during the current study.
